# Identification of major quantitative trait loci and candidate genes for seed weight in soybean

**DOI:** 10.1007/s00122-023-04299-w

**Published:** 2023-01-23

**Authors:** Mengge Xu, Keke Kong, Long Miao, Jianbo He, Tengfei Liu, Kai Zhang, Xiuli Yue, Ting Jin, Junyi Gai, Yan Li

**Affiliations:** National Key Laboratory of Crop Genetics and Germplasm Enhancement, National Center for Soybean Improvement, Key Laboratory for Biology and Genetic Improvement of Soybean (General, Ministry of Agriculture), Jiangsu Collaborative Innovation Center for Modern Crop Production, Nanjing Agricultural University, Nanjing, China

## Abstract

**Key message:**

Four major quantitative trait loci for 100-seed weight were identified in a soybean RIL population under five environments, and the most likely candidate genes underlying these loci were identified.

**Abstract:**

Seed weight is an important target of soybean breeding. However, the genes underlying the major quantitative trait loci (QTL) controlling seed weight remain largely unknown. In this study, a soybean population of 300 recombinant inbred lines (RILs) derived from a cross between PI595843 (PI) and WH was used to map the QTL and identify candidate genes for seed weight. The RIL population was genotyped through whole genome resequencing, and phenotyped for 100-seed weight under five environments. A total of 38 QTL were detected, and four major QTL, each explained at least 10% of the variation in 100-seed weight, were identified. Six candidate genes within these four major QTL regions were identified by analyses of their tissue expression patterns, gene annotations, and differential gene expression levels in soybean seeds during four developmental stages between two parental lines. Further sequence variation analyses revealed a C to T substitution in the first exon of the *Glyma.19G143300*, resulting in an amino acid change between PI and WH, and thus leading to a different predicted kinase domain, which might affect its protein function. *Glyma.19G143300* is highly expressed in soybean seeds and encodes a leucine-rich repeat receptor-like protein kinase (LRR-RLK). Its predicted protein has typical domains of LRR-RLK family, and phylogenetic analyses reveled its similarity with the known LRR-RLK protein *XIAO* (*LOC_Os04g48760*), which is involved in controlling seed size. The major QTL and candidate genes identified in this study provide useful information for molecular breeding of new soybean cultivars with desirable seed weight.

**Supplementary Information:**

The online version contains supplementary material available at 10.1007/s00122-023-04299-w.

## Introduction

Soybean [*Glycine max* (L.) Merr.] is an economically important crop, which not only provides vegetable protein and edible oil for human and animals (Lu et al. 2016), but also plays an important role in biofuel production and soil fertility improvement (Kulkarni et al. 2016). The demand for soybean continues to increase, especially in China, and thus, improving soybean yield is still the main goal for soybean breeding. The 100-seed weight is an important yield-related trait, and also one of the targets under selection during soybean domestication (Duan et al. 2022; Goettel et al. 2022). Therefore, identification of the genetic loci and candidate genes for the seed weight is important for soybean genetic improvement (Liang et al. 2005).

There is great variation in soybean 100-seed weight, ranging from 7.30 to 23.60 g and from 5.64 to 34.80 g in the germplasm collections from the USA and China, respectively (Zhang et al. 2016; Zhao et al. 2019). The quantitative trait loci (QTL) controlling 100-seed weight have been identified by genome-wide association studies (GWAS) (Fang et al. 2017; Hao et al. 2012; Karikari et al. 2020; Li et al. 2019b; Zhang et al. 2016, 2015b) and linkage mapping (Han et al. 2012; Hoeck et al. 2003; Karikari et al. 2019; Kato et al. 2014; Kim et al. 2010; Li et al. 2020; Liu et al. 2007; Lu et al. 2017; Panthee et al. 2005; Teng et al. 2009; Yan et al. 2017; Yang et al. 2019). However, many QTL for 100-seed weight were mapped to relatively large genomic regions, due to low-density markers, small mapping population size, or lack of recombination, which causes difficulties to identify candidate genes in these regions. Furthermore, just few major and/or stable QTL for 100-seed weight across multiple environments have been reported, which are important for soybean breeding program via marker-assisted selection (MAS).

The genes underlying the QTL of soybean 100-seed weight are still largely unknown. Just few genes related to seed weight/size have been verified in soybean. Overexpression of *GmCYP78A72*, a gene encoding a cytochrome P450 protein, increased seed weight in transgenic lines (Adamski et al. 2009; Zhang et al. 2016, 2015a; Zhao et al. 2016). Another gene, soybean *GA20OX* (*Glyma07g08950*, encoding gibberellin 20 oxidase 2), was identified through transcriptome analysis and was found to be able to enhance seed size/weight by its ectopic expression in transgenic Arabidopsis plants (Lu et al. 2016). Ectopic expression of *PP2C-1* (*Glyma17g33690*, encoding a putative phosphatase 2C protein) from wild soybean ZYD7 also significantly enhanced the seed weight/size of Arabidopsis (Lu et al. 2017). *GmSWEET10a* and *GmSWEET10b* (*Glyma.15G049200* and *Glyma.08G183500*), both encoding a member of the SWEET family of sugar transporters, control the sugar allocation from seed coat to embryo to affect the seed weight/size and seed oil content in soybean (Wang et al. 2020). Down-regulation of *GmBS1* (*Glyma10g38970*, encoding a TIFY transcription factor) leads to significant increases in the sizes of soybean organs, including leaf and seed (Ge et al. 2016). *GmKIX8-1* (*Glyma.17G112800*, encoding a KIX domain-containing protein), located within the major 100-seed weight QTL of *qSw17-1*, has been verified for its function in regulating cell proliferation (Nguyen et al. 2021), specifically, the loss of function of *GmKIX8-1* resulted in increased sizes of aerial soybean organs, such as seeds and leaves. Recently, the natural variations of three genes were found associated with soybean seed size/weight, including *GmST1*, *GmST05*, and *POWR1* (Duan et al. 2022; Goettel et al. 2022; Li et al. 2022a). Both *GmST1* (*Glyma.08g109100*, encoding a UDP-D-glucuronate 4-epimerase) and *GmST05* (*Glyma.05G244100*, encoding a member of the FT and TFL1 family of phosphatidylethanolamine-binding protein) function as positive regulators of seed thickness, seed length, seed width, and 100-seed weight in soybean (Duan et al. 2022; Li et al. 2022a). *POWR1* (*Glyma.20G085100*), encoding a CCT (CONSTANS, CONSTANS-like, TOC1) motif-containing protein, was found to have pleiotropic effects on seed weight/yield, oil and protein content (Goettel et al. 2022). Considering the large genetic variation and many QTL for 100-seed weight in soybean have been reported (https://www.soybase.org), more genes especially the ones within the major QTL related to soybean seed weight need to be discovered.

To further identify the major and/or stable QTL and candidate genes for 100-seed weight in soybean, a population of 300 recombinant inbred lines (RILs) derived from a cross between PI595843 (PI) and WH was genotyped by using the whole genome resequencing, and phenotyped under five environments. The major and stable QTL as well as their candidate genes for 100-seed weight were identified, which would be useful in the genetic improvement of 100-seed weight in soybean.

## Materials and methods

### Plant materials

The soybean RIL population (NJPW-RIL) of 300 lines, developed through single seed descent method, from the cross of PI595843 (PI, a cultivar originated from Ohio, USA) and WH (a landrace originated from Anhui province, China), was obtained from the National Center for Soybean Improvement (Nanjing, China).

### Experimental design and measurement of seed weight

The two soybean parental accessions and 300 RILs were grown in a randomized complete block design (RCBD), under five environments (with three replications within each environment) across four years (normal summer growing season). The field experiments were conducted in three locations, including Liuhe Experimental Station (abbreviated as LH) in Nanjing, Jiangsu Province (Latitude 32°11′ N; Longitude 118°34′ E), Jiangpu Experimental Station (abbreviated as JP), Nanjing, Jiangsu Province (Latitude 33°03′ N; Longitude 118°63′ E), and Dangtu Experimental Station (abbreviated as DT), Maanshan, Anhui Province (Latitude 32°87′ N; Longitude 117°56′ E). The five environments were designated as year-location: 2014LH, 2015JP, 2015DT, 2018DT, and 2019DT. The soybean lines were planted in 1-m-length rows, with a distance of 10 cm between plants and a row spacing of 50 cm. Mature seeds were harvested for each line and dried to a stable weight under 35–40 °C. For each sample, the weight of 100 randomly selected healthy mature dry seeds (using a seed counting plate) was measured by an electronic balance, and the average value of three technical repeats was used as its 100-seed weight (g) value.

### Resequencing and genotyping of the NJPW-RIL population

The 300 individuals of NJPW-RIL (F_2:10_ generation) and two parents were grown in a greenhouse. After three weeks, approximately 1 g of fresh leaves was obtained for extracting the genomic DNA using the cetyltrimethylammonium bromide (CTAB) method (Doyle and Doyle 1990). About 1 mg of DNA for each sample was sheared into approximately 350–400 bp DNA fragments by a sonicator (Covaris, Massachusetts, USA). TruSeq Library Construction Kit was used to prepare the resequencing library, according to the manufacturer’s protocol. The DNA fragments were end-repaired, tailed with “A” nucleotides and ligated to Illumina paired-end sequencing adapters. Then, the paired-end sequencing libraries were sequenced on an Illumina HiSeqX high-throughput sequencing platform for PE150 pair-end sequencing.

The paired-end sequencing adapters, raw reads containing ≥ 10% unidentified nucleotides (N), low-quality (Q-score ≤ 5) reads, and DNA of other sources were all filtered out to obtain the high-quality clean data. The clean data were then aligned to the soybean reference genome (Schmutz et al. 2010) Williams 82 (*Glycine max* v2.1 genome) by using Burrows-Wheeler Aligner (BWA) (Version: 0.6.1-r104) based on the default parameters (Li and Durbin 2009). Then, the alignment files were converted to BAM files and sorted by Sequence Alignment/Map tools (SAMtools) (Li et al. 2009). Finally, the uniquely mapped reads were used for variation detection.

The Genome Analysis Toolkit (GATK) software (McKenna et al. 2010) was applied for single nucleotide polymorphisms (SNP) calling in NJPW-RILs and two parents. To reduce false-positive SNPs caused by sequencing errors, the SNP base support numbers for each parent and the offspring were set as ≥ 5 and ≥ 3, respectively. ANNOVER software (Wang et al. 2010) was used to annotate SNPs based on the reference genome. Only the bi-allelic SNPs were further screened. We filtered out the abnormal bases and selected markers to cover ≥ 75% of lines in soybean NJPW-RIL population. The SNPs deviated from the expected Mendelian segregation ratio 1:1 (*P* < 0.001 for Chi-square test) were excluded to obtain the high-quality SNPs. The consecutive SNPs were scanned with a window size of 15 SNPs and a step length of 1 cM by using a sliding window approach (Han et al. 2016; Huang et al. 2009) to identify the recombination breakpoints, which were identified as a transition from one genotype to the other. The interval with the same parental genotype in the RIL population was considered as a bin.

### Construction of genetic linkage map

The bins were used as genetic markers for the construction of a linkage map for the NJPW-RIL population by using JoinMap 4.0 software (Van Ooijen 2006). The genetic distance between bin markers was calculated by using the Kosambi mapping function (Kosambi 1944). The bin markers were assigned to chromosomes by setting a minimum logarithm of odds (LOD) score of 3.0. Finally, a genetic map was displayed by using R/qtl (Arends et al. 2010).

### QTL analysis

QTL analysis was performed using the composite interval mapping (CIM) method (Zeng 1994) in the WinQTLCart 2.5 software (Wang et al. 2012; Yang et al. 2007). The mean values of 100-seed weight under single environment and five environments were used as the phenotypic data. The LOD threshold was calculated by 1000 permutation tests with a significance level of 0.05 (Churchill and Doerge 1994) to declare a QTL. The confidence interval of each QTL was estimated using 1-LOD. We followed the nomenclature (McCouch et al. 1997) with modifications to name the QTL in this study; for example, *qSw-2-1*, *q* represents the QTL; *Sw* represents the 100-seed weight; *-2* represents chromosome 2; *-1* represents the first QTL on that chromosome. If the QTL in different environments shared the same or overlapped confidence intervals and had the same direction (positive or negative) of additive effects, they were considered as the same QTL. The major QTL was defined in this study when it explained at least 10% of the phenotypic variation.

### Identification of potential candidate genes for 100-seed weight

The potential candidate genes for 100-seed weight within the major QTL were identified through the following steps: (1) the gene IDs and annotations within the physical interval of the major QTL were downloaded from the soybean genome Williams 82 (*Glycine max* v2.1 genome) (https://www.soybase.org). (2) the RNA-seq data (fragments per kilobase of transcript per million mapped reads, FPKM) of these genes in different soybean tissues were downloaded from Phytozome (https://phytozome-next.jgi.doe.gov/), and the genes with higher expression levels in soybean seeds (ΔFPKM = FPKM_seed_ − FPKM_mean_ ≥ 10) were selected for further analysis. The FPKM values were used to draw the heatmaps by using MeV 4.9.0 software (https://sourceforge.net/projects/mev-tm4/files/mev-tm4/). (3) those genes with higher expression levels in soybean seeds and have the functional annotations in the known signaling pathways controlling seed size/weight, including ubiquitin–proteasome pathway, G-protein signaling, mitogen-activated protein kinase (MAPK) signaling, phytohormones and transcriptional regulatory factors (Li and Li 2016; Li et al. 2019a), were identified as potential candidate genes for soybean 100-seed weight, which were then subjected to expression and sequence variation analyses.

### Quantitative real-time (qRT)-PCR

The qRT-PCR was employed to compare the expression levels of the potential candidate genes in the seeds of two parental lines, PI and WH, at different developmental stages. The soybean varieties PI and WH were planted at Dangtu Experimental Station, Maanshan, Anhui Province in 2019. Then, the seeds were sampled on the 10, 20, 30 and 40 days after flowering (DAF) with three biological replications. The total RNA was isolated using a Plant RNA Extract Kit (TianGen, Beijing, China) according to the manufacturer’s instructions. The first-strand cDNA was synthesized by using PrimeScriptTM RT Master Mix (Perfect Real Time) (Vazyme, China). The gene specific primers (Supplementary Table 1) were designed at NCBI website and synthesized at GenScript (Nanjing, China). The reactions of qRT-PCR were performed using the SYBR Green Master Mix (Vazyme, China) according to the manufacturer’s protocol, on a LightCycler 480 System (Roche, Penzberg, Upper Bavaria, Germany). The qRT-PCR amplification conditions were 95 °C for 30 s followed by 40 cycles of 95 °C for 10 s, 58 °C for 30 s. The *GmUKN1* (*Glyma.12g020500*, GenBank accession no. NM_001254696.2) was used as the reference gene (Hu et al. 2009) to normalize the relative expression levels of test genes. The relative expression level was calculated by 2^−△△CT^ methods (Livak and Schmittgen 2001). Each sample has three biological and three technical replications.

### Sequence variation analyses and protein structure prediction

To further compare the sequence variation of the candidate genes, the full-length coding sequences (CDS) of the candidate genes were amplified using the cDNA from PI, WH and 60 RILs with extreme phenotypes as templates, and the gene-specific primers (Supplementary Table 1) were designed by NCBI and synthesized at GenScript (Nanjing, China). The amplicons were sequenced at TSINGKE (Beijing, China). We then used the public available data from http://www.mbkbase.org/ (Peng et al. 2020) to validate the allelic effect of the candidate gene on seed weight in more soybean accessions. The sequence variations of the candidate gene were downloaded from http://www.mbkbase.org/soybean/genotype/byLocus/GmaxG00051878. The phenotypes were downloaded from http://www.mbkbase.org/soybean/germplasm. The sequences were aligned and compared using ClustalX 2.1 software (Larkin et al. 2007).

The protein domains were predicted by SMART (http://smart.embl-heidelberg.de/) (Letunic et al. 2021). The three-dimensional protein structures were predicted by Phyre2 (http://www.sbg.bio.ic.ac.uk/phyre2/html/page.cgi?id=index) (Kelley et al. 2015).

### Phylogenetic analysis

The sequences used for phylogenetic analysis were obtained from NCBI (https://www.ncbi.nlm.nih.gov/). The phylogenetic tree was constructed by using MEGA 6.0 (Tamura et al. 2013) based on the neighbor-joining method with 1000 bootstraps. The multiple sequences were aligned and compared using ESPript 3.0 (https://espript.ibcp.fr/ESPript/cgi-bin/ESPript.cgi) (Robert and Gouet 2014).

### Statistical analyses

The descriptive statistics and analysis of variance (ANOVA) of the 100-seed weight across five environments were conducted using the programs of MEANS and PROC GLM by SAS 9.4 (SAS Institute, Cary, NC). The heritability was estimated by the equation: ${h}^{2}={\sigma }_{g}^{2}/({\sigma }_{g}^{2}+{\sigma }_{e}^{2}/r)\times 100\mathrm{\%}$ and ${h}^{2}={\sigma }_{g}^{2}/({\sigma }_{g}^{2}+{\sigma }_{ge}^{2}/n+{\sigma }_{e}^{2}/nr)\times 100\mathrm{\%}$ for a single environment and the multiple environments, respectively; where ${\sigma }_{g}^{2}$, ${\sigma }_{ge}^{2}$ and ${\sigma }_{e}^{2}$ represent genotypic variance, variance of the genotype-by-environment interaction and random error variance, respectively; *n* is the number of environments and *r* is the number of replications (Nyquist and Baker 1991). The genotypic coefficient of variation (*GCV*) for the 100-seed weight was calculated as *GCV* = ${\sigma }_{g}/\mu$, where ${\sigma }_{g}$ is the genetic standard deviation, and $\mu$ is the mean value of 100-seed weight under each environment (Nyquist and Baker 1991). The differences between the groups were analyzed by using two-tailed Student’s *t*-test and two-sided Wilcoxon test.

## Results

### Phenotypic variation of 100-seed weight in the NJPW-RIL population

**Fig. 1:**
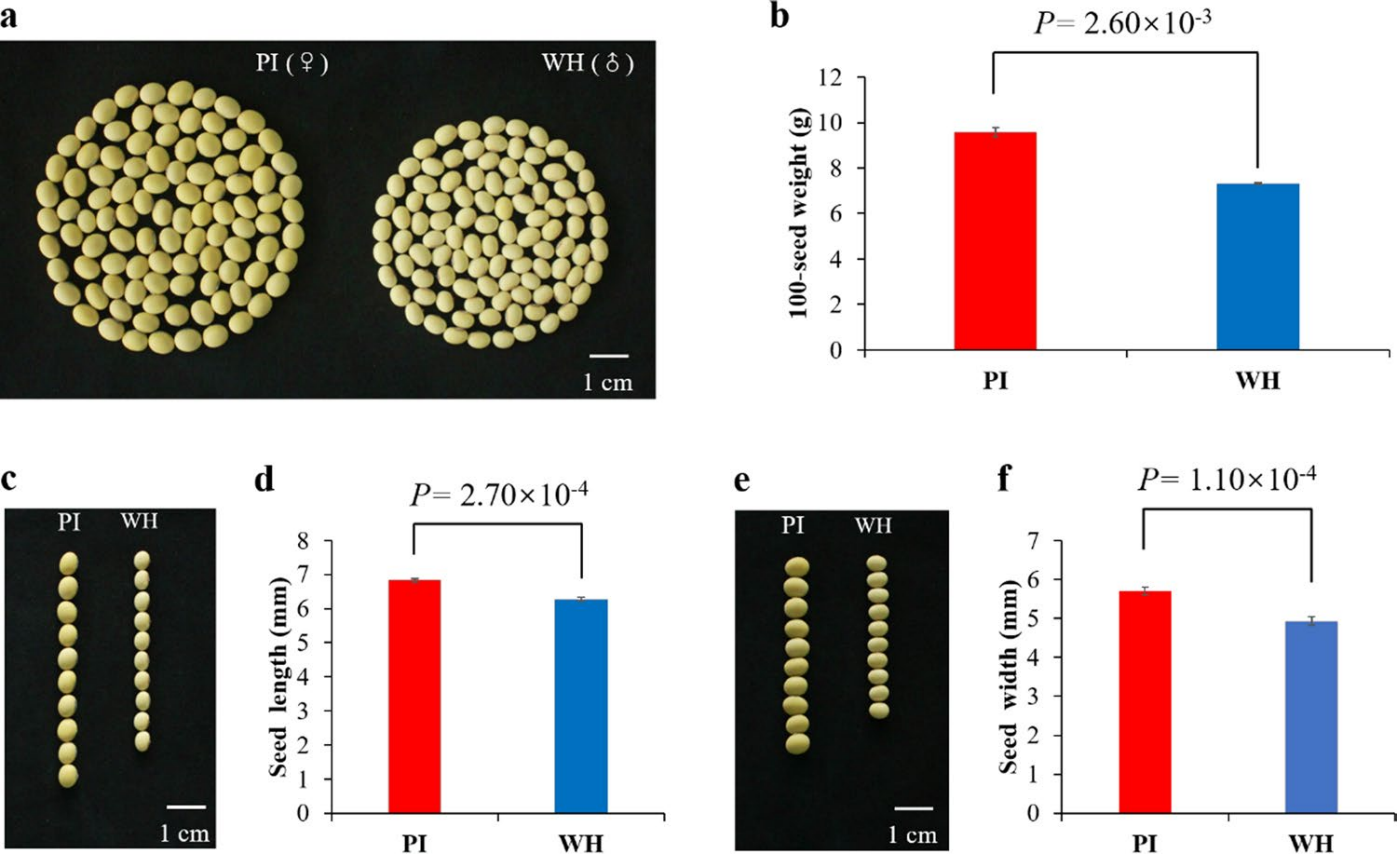
Seed traits of the two parental soybean accessions PI and WH. **a** Seed morphology of PI and WH. Scale bar, 1 cm. **b** Statistical analysis of the 100-seed weight of PI and WH. **c** Seed length of PI and WH. Scale bar, 1 cm. **d** Statistical analysis of the seed length of PI and WH. **e** Seed width of PI and WH. Scale bar, 1 cm. **f** Statistical analysis of the seed width of PI and WH. The photograph and phenotypic data of 100-seed weight, seed length and seed width were obtained under 2019DT environment. All data and error bars in charts represent mean ± standard deviation of three replications (*n* = 100 × 3 for 100-seed weight; *n* = 10 × 3 for seed length and seed width). Student’s *t*-tests (two-tail) were used to compare the significant differences between PI and WH

There are significant differences in seed traits between the two parental soybean accessions PI and WH (Fig. 1a-f), including 100-seed weight, seed length and width. The phenotypic variation of 100-seed weight among the NJPW-RILs and the two parental accessions across five environments (2014LH, 2015DT, 2015JP, 2018DT, 2019DT), as well as the mean values are shown in Table 1 and Supplementary Table 2. The 100-seed weight of the NJPW-RIL population ranged from 8.91 g to 21.57 g based on average values over five environments, indicating there is a large variation in this RIL population (Supplementary Fig. 1a–f). The heritability of 100-seed weight was 91.83% across five environments, suggesting that the phenotypic variation in 100-seed weight is mainly controlled by genetic variation (Table 1). The genotypes/lines, environments and their interactions had significant effects on 100-seed weight in the NJPW-RIL population (Table 2).

**Table 1:** Descriptive statistics of 100-seed weight in the NJPW-RIL population under multiple environments

Environment	Parents (g)		NJPW-RILs (g)								
	PI	WH	Minimum	Maximum	Range	Means ± SD	CV (%)	Skewness	Kurtosis	*GCV* (%)	*h*^2^ (%)
2014LH	10.05	8.43	8.77	23.38	14.61	13.22 ± 1.93	14.62	0.86	2.51	14.12	97.47
2015DT	9.42	8.35	9.28	24.43	15.15	12.88 ± 1.83	14.24	1.38	5.42	15.27	96.07
2015JP	12.17	8.75	7.71	20.06	12.35	11.61 ± 1.67	14.35	1.22	3.87	11.69	94.45
2018DT	11.73	8.20	7.81	–	9.58	11.25 ± 1.60	14.20	0.46	0.12	10.68	83.42
2019DT	10.65	9.07	6.71	20.30	13.59	12.17 ± 1.97	16.16	0.40	1.04	13.37	86.13
MEAN	10.80	8.56	8.91	21.57	12.66	12.24 ± 1.55	12.70	1.08	4.01	11.93	91.83

2014LH, experiment at Liuhe in 2014; 2015DT, experiment at Dangtu in 2015; 2015JP, experiment at Jiangpu in 2015; 2018DT, experiment at Dangtu in 2018; 2019DT, experiment at Dangtu in 2019; MEAN, the average values of 100-seed weight across five environments of 2014LH, 2015DT, 2015JP, 2018DT and 2019DT. “–”, the data were missing for the line with maximum 100-seed weight. *GCV*, genotypic coefficient of variation. *h*^*2*^, heritability

**Table 2:** Analysis of variance for 100-seed weight in the NJPW-RIL population

Variation Source	DF	SS	MS	*F* value	*P* value
Genotype	299	9468.94	31.67	24.22	< .0001
Environment	4	1849.84	462.46	353.65	< .0001
Replications (Environment)	10	84.80	8.48	6.48	< .0001
Genotype × Environment	1190	3459.55	2.91	2.22	< .0001
Error	2762	3611.83	1.31		

Environment, five independent experiments were performed in 2014LH, 2015DT, 2015JP, 2018DT and 2019DT. DF, Degree of Freedom. SS, Sum of Squares. MS, Mean Square

### Genetic linkage map of NJPW-RIL population

The 300 NJPW-RILs and two parental lines were genotyped by whole genome resequencing. A total of 12,648,198,300 bp (12.65 Gb) and 11,022,993,600 bp (11.02 Gb) raw data were obtained for PI and WH, respectively, with an average coverage of approximately 10 × depths. The quality of sequencing data for two parents was high, with effective rate (%) ≥ 99.79%, Q20 ≥ 97.22%, Q30 ≥ 92.36%, and error rate ≤ 0.03% (Supplementary Table 3). Subsequently, a total of 862.70 Gb of Illumina paired-end read sequence data was generated for 300 NJPW-RILs with a mean depth of about 2 × , and the quality reached Q20 ≥ 93%, Q30 ≥ 85%, and error rate ≤ 0.05%.

**Fig. 2:**
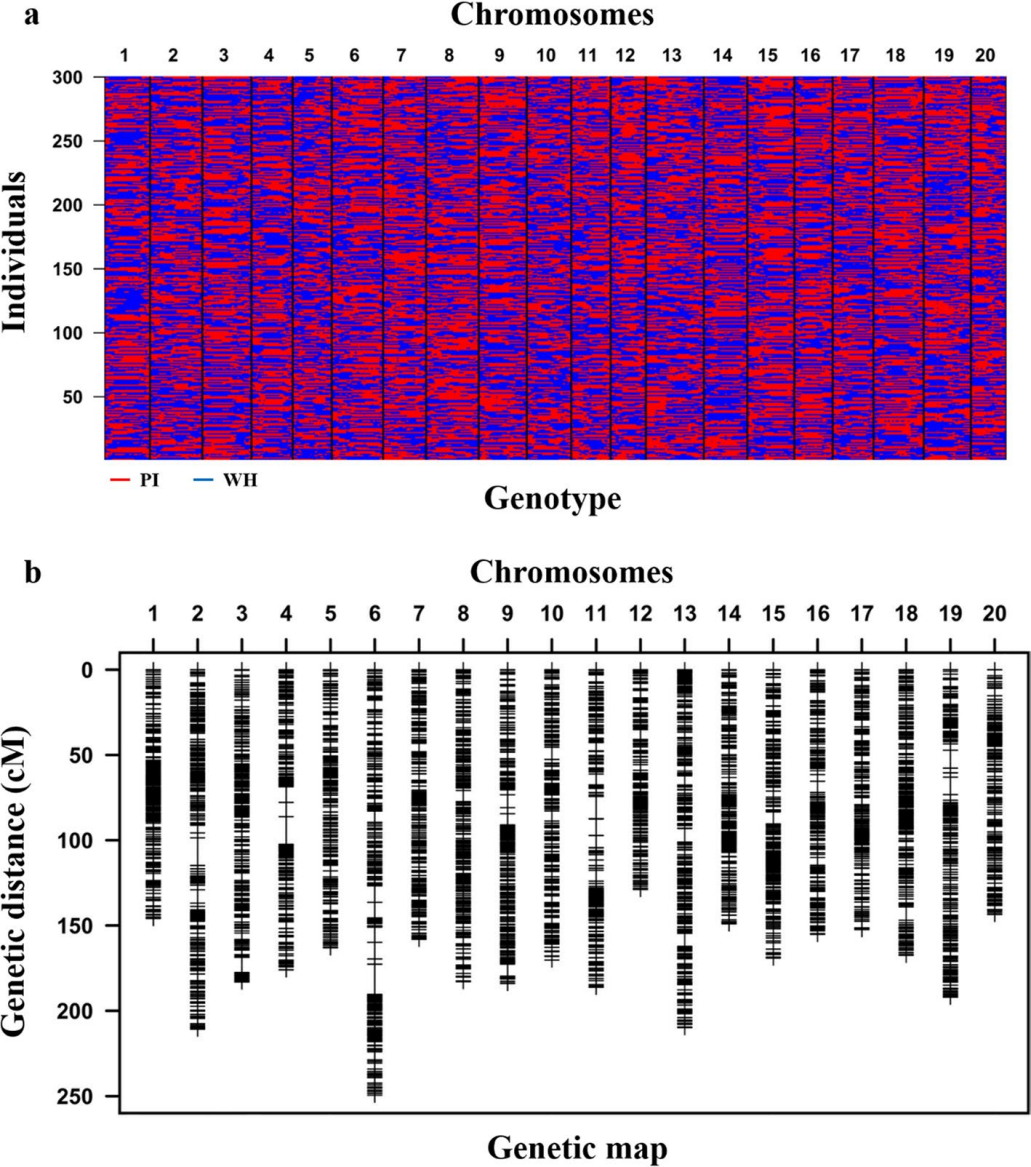
Genotyping map and genetic map constructed from resequencing data of the NJPW-RIL population. **a** The genotype of 4702 bins based on the recombination breakpoints identified in 300 NJPW-RILs derived from the cross of PI and WH. Each horizontal line represents a single RIL across 20 soybean chromosomes. Red and blue bars represent the parental genotypes of PI and WH, respectively. **b** Distribution and genetic distance of bin markers on 20 soybean chromosomes in the NJPW-RIL population. The horizontal black lines on each chromosome represent bin markers

After removing the low-quality reads, the clean data were aligned against the soybean reference genome Williams 82 (*Glycine max* v2.1 genome). The coverage (1 ×) is 98.12% and 96.98% for PI and WH (Supplementary Table 4), respectively, and the average mapping rate of NJPW-RILs is 81.89% (Supplementary Fig. 2). A total of 1,673,234 SNPs showed polymorphism between PI and WH. After filtering, 1,161,784 high-quality SNPs were used to identify the recombination breakpoints, and a total of 4702 bins were identified and genotyped for 300 RILs (Fig. 2a). Finally, a genetic linkage map of 4702 bins (Supplementary Table 5) on 20 linkage groups/chromosomes was constructed (Fig. 2b). Chromosome 13 had the maximum number of bin markers (302 bins), whereas chromosome 12 contained the minimum number (184) of bins (Supplementary Table 5). The average genetic distance between two adjacent bins on 20 chromosomes was 0.74 cM, which corresponds to approximately 200 kb in physical distance, indicating that the resolution of this map is sufficient for QTL mapping in this RIL population.

### The QTL identified for 100-seed weight in the soybean NJPW-RIL population

**Fig. 3:**
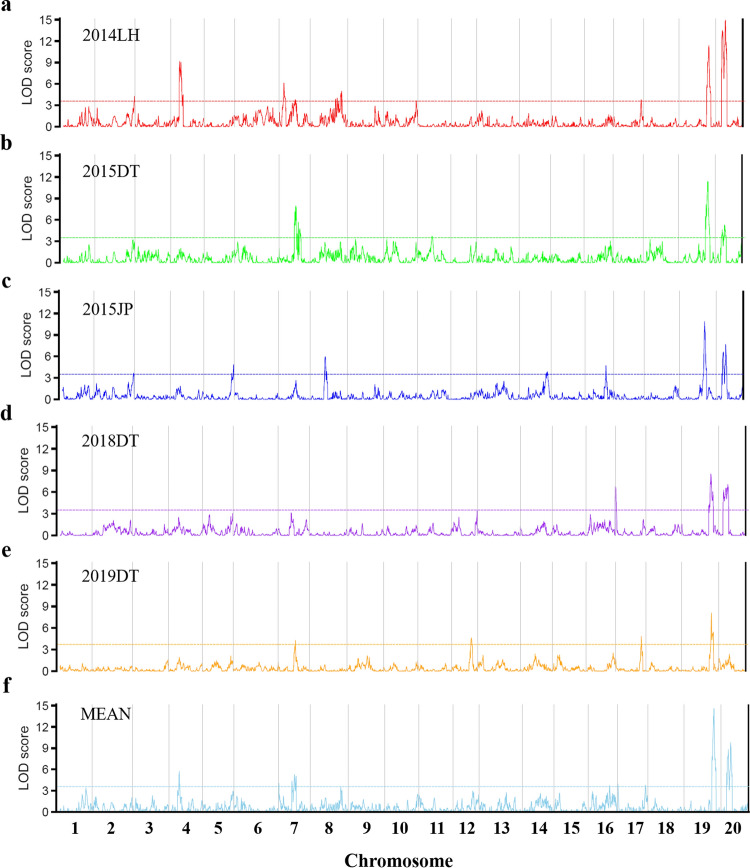
The quantitative trait loci (QTL) for 100-seed weight identified in the NJPW-RIL population under multiple environments. **a** 2014LH, **b** 2015DT, **c** 2015JP, **d** 2018DT, **e** 2019DT and **f** MEAN represent the environments of 2014Liuhe, 2015Dangtu, 2015Jiangpu, 2018Dangtu, 2019Dangtu, and the mean value of 100-seed weight across five environments, respectively. LOD, logarithm of odds; the horizontal dotted lines represent LOD thresholds calculated from 1000-permutation tests (significance level of 0.05) by using the CIM model in WinQTLCart2.5 Software, which were 3.60, 3.50, 3.50, 3.50, 3.70 and 3.60 for 2014LH, 2015DT, 2015JP, 2018DT, 2019DT and MEAN (the mean value of 100-seed weight value across five environments), respectively

A total of 38 QTL for 100-seed weight were detected by CIM procedure in the NJPW-RIL population under multiple environments, which were distributed on chromosomes 2, 4, 5, 7, 8, 10, 11, 12, 14, 16, 17, 19 and 20 (Fig. 3a–f, Supplementary Fig. 3 and Supplementary Table 6), with LOD scores ranging from 3.58 to 14.92, and explained 3.01% to 15.03% of the phenotypic variation (*R*^2^). Among them, 12 QTL were identified in at least two environments. Four major QTL had large-contribution to the phenotypic variation (*R*^2^ ≥ 10% for each one), including *qSw-19-1*, *qSw-19-5*, *qSw-20-2* and *qSw-20-3*. The first major QTL, *qSw-19-1* on chromosome 19, was detected in the 2015JP environment, which accounted for 11.60% of the phenotypic variation in 100-seed weight. The second major QTL, *qSw-19-5*, was identified in three environments (2014LH, 2015DT, 2019DT) and by the mean values across five environments (MEAN), which explained 9.52% to 13.43% of the phenotypic variation. The other two major QTL, *qSw-20-2* and *qSw-20-3*, were detected in four environments (2014LH, 2015DT, 2015JP, 2018DT) and by the mean values across five environments (MEAN), accounting for 4.15%—13.33% and 5.08—15.03% of the phenotypic variation, respectively. Three out of the four major QTL, including *qSw-19-5*, *qSw-20-2* and *qSw-20-3*, were detected in multiple environments, which therefore are considered as the stable major QTL for 100-seed weight in the NJPW-RIL population (Supplementary Table 6).

Among the 38 100-seed weight QTL detected in the NJPW-RIL population, four were identified in this study for the first time, including *qSw-7-1*, *qSw-10-1*, *qSw-14-1* and *qSw-16-1*, which could be novel QTL (Supplementary Table 6). The other 34 QTL co-localized with the previously reported 100-seed weight QTL, but had a smaller physical interval (Supplementary Table 6). Among the 38 QTL, the alleles with positive additive effect (increasing 100-seed weight) of 32 QTL were from the female parent PI with larger seed weight, while the positive alleles of *qSw-4-1*, *qSw-4-2*, *qSw-4-3*, *qSw-7-8*, *qSw-12-1* and *qSw-14-1* came from the other parental line WH (Supplementary Table 6).

### Candidate genes for 100-seed weight in the major QTL intervals

Within the genomic region of the four major QTL (*qSw-19-1*, *qSw-19-5*, *qSw-20-2* and *qSw-20-3*), a total of 65, 92, 292 and 147 annotated genes were found, respectively. Among these genes, 34 genes with higher expression levels in soybean seeds than other tissues were considered as the potential candidate genes (Supplementary Fig. 4). Then, six out of 34 genes, which have the functional annotations in the known signaling pathways controlling seed size/weight (Li and Li 2016; Li et al. 2019a), were identified as candidate genes for soybean 100-seed weight for further analyses (Supplementary Table 7).

**Fig. 4:**
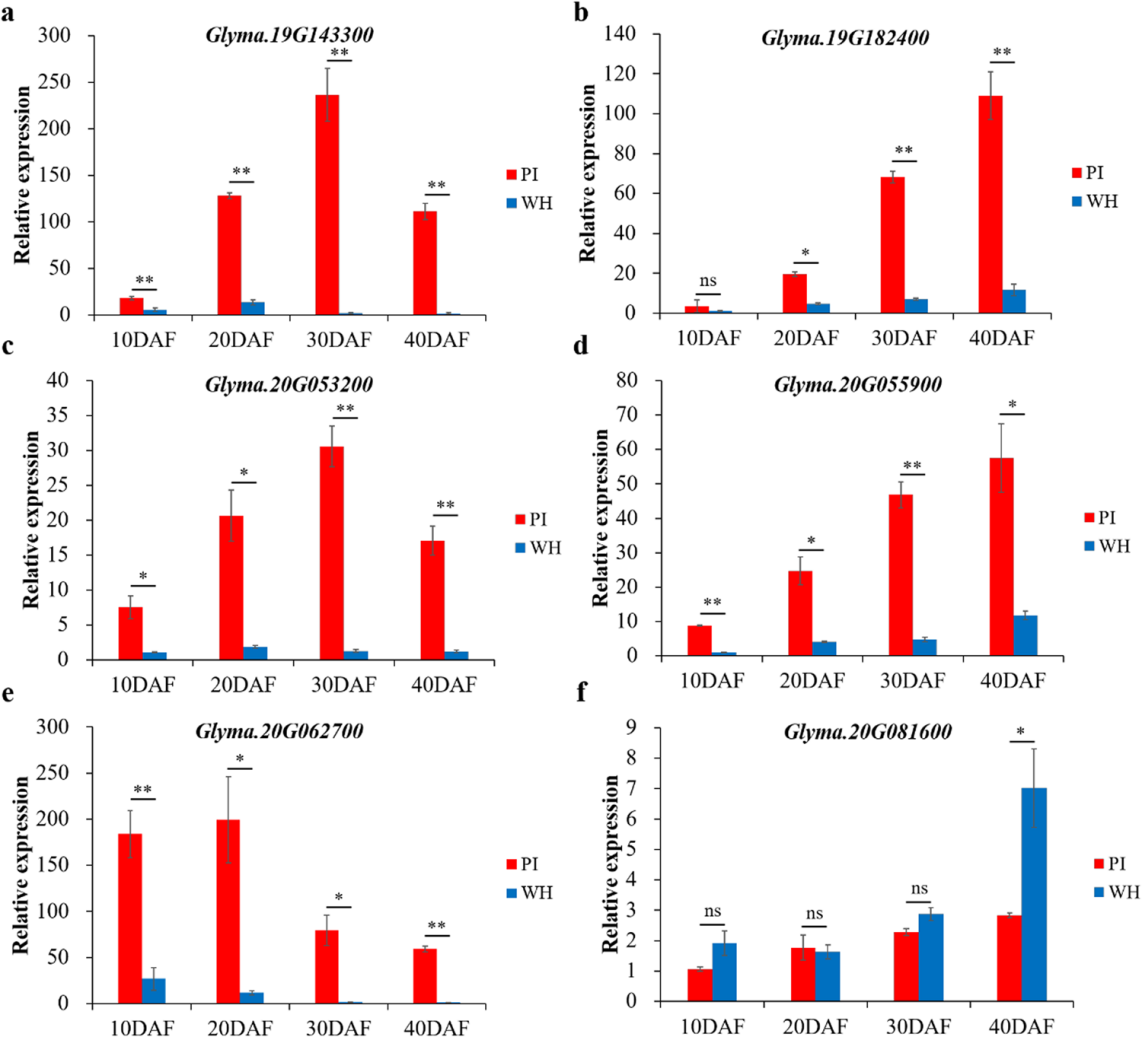
Relative expression levels of six candidate genes in the seeds of two parental soybean accessions PI and WH at different developmental stages. Relative expression levels of six candidate genes, including *Glyma.19G143300* **a** *Glyma.19G182400* **b** *Glyma.20G053200* **c** *Glyma.20G055900* **d** *Glyma.20G062700* **e** and *Glyma.20G081600* **f**, in the seeds of two parental lines PI (larger seed) and WH (smaller seed) at four developmental stages of 10, 20, 30, and 40 DAF (days after flowering). *GmUKN1* (*Glyma.12G02500*) was used as an internal control. The data represent the mean ± standard deviation (*n* = 3 × 3 = 9). * and ** represent significant difference in the relative expression level between PI and WH at 0.05 and 0.01 level, respectively; ns, not significant (Student’s *t*-test, two-tail)

The expression levels of these six candidate genes in soybean seeds at different developmental stages were analyzed by qRT-PCR using the gene specific primers (Supplementary Table 1). As shown in Fig. 4a-e, the relative expression levels of five genes, including *Glyma.19G143300*, *Glyma.19G182400*, *Glyma.20G053200*, *Glyma.20G055900*, and *Glyma.20G062700*, were significantly higher in the seeds of the parental accession PI (larger seeds) than WH (smaller seeds), at four developmental stages of 10, 20, 30, and 40 DAF. Whereas the expression level of *Glyma.20g081600* only showed higher expression levels in the seeds of WH than PI at 40 DAF (Fig. 4f). Since these six genes all showed differential expression in seeds between the two parental lines, they were subjected to further sequence analyses.

### Sequence variation of the candidate genes for 100-seed weight

**Fig. 5:**
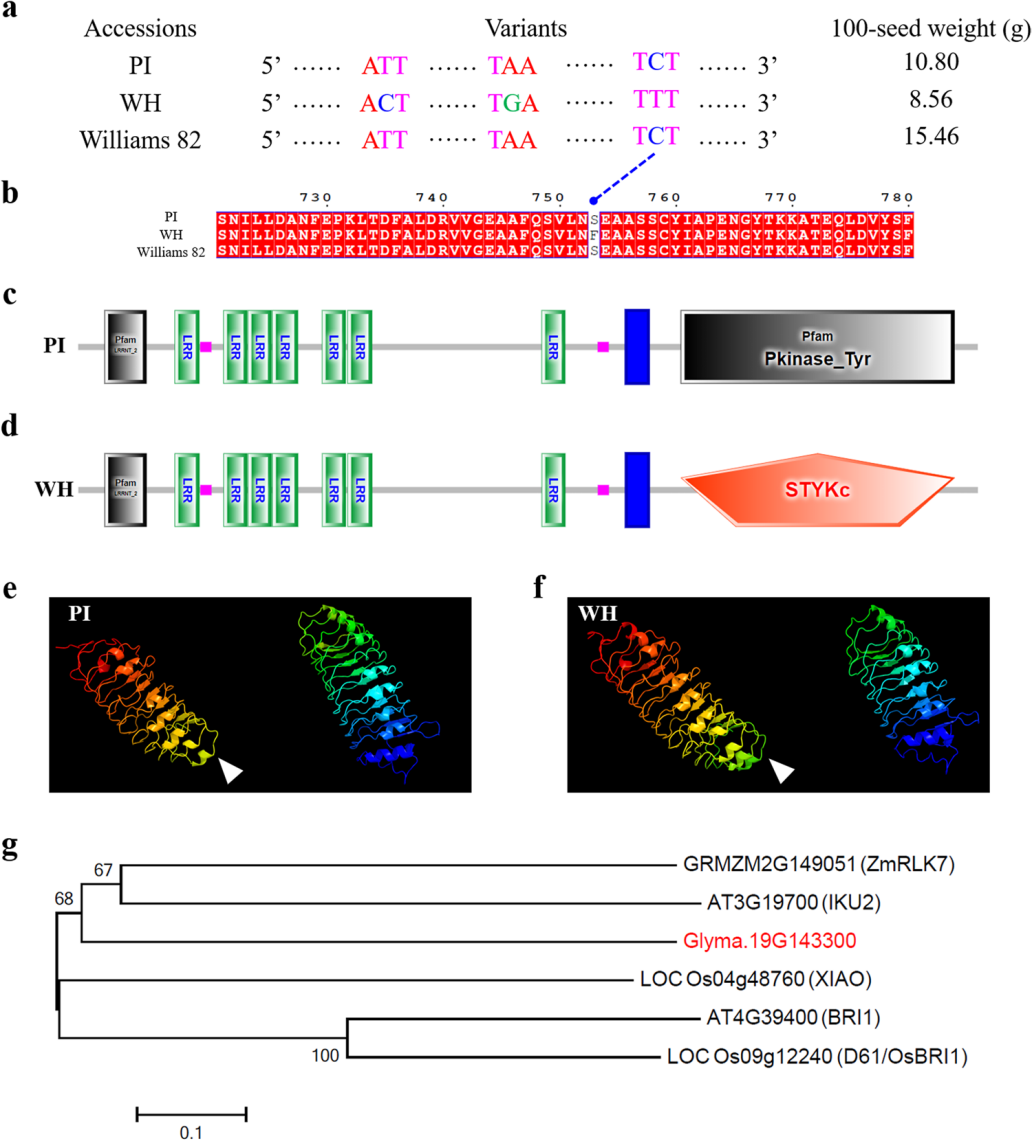
Sequence analyses of Glyma.19G143300 and its predicted protein structure. **a** Polymorphisms in the coding region of *Glyma.19G143300* between the two parental lines of soybean RIL population and the reference genome sequence of Williams 82. **b** The amino acid change of S (serine) to F (phenylalanine) due to the SNP polymorphism in the coding region of *Glyma.19G143300* as shown in **a**. **c**, **d** The predicted protein structure of Glyma.19G143300 in PI and WH, respectively. The first gray boxes represent LRRNT_2 domains (leucine-rich repeats at the N terminus), the green boxes represent LRR (tandem leucine-rich repeats) domains, the blue boxes represent transmembrane regions, and the boxes at the end represent the kinase domains of Pkinase_Tyr domain in **c** (gray box) and STYKc domain in **d** (orange box). **e**, **f** The three-dimensional structure of Glyma.19G143300 protein in PI and WH, respectively. The white arrows indicate the difference between PI and WH. **g** Phylogenic tree of Glyma.19G143300 and the known leucine-rich repeat receptor-like kinase (LRR-RLK) proteins. The tree was constructed using MEGA version 6.0. The numbers on the branches indicate the 1000 bootstrap values. Scale bar unit, divergence distance. The figure was generated using the full-length amino acid sequences of the proteins, including AT3G19700 and AT4G39400 from *Arabidopsis thaliana*, LOC_Os04g48760 and LOC_Os09g12240 from *Oryza sativa*, GRMZM2G149051 from *Zea mays* and Glyma.19G143300 from *Glycine max*

The sequence variations of above six genes were first investigated by comparing the resequencing data of PI and WH, and we only found sequence polymorphisms in three genes, including *Glyma.19G143300*, *Glyma.19G182400* and *Glyma.20g081600* (Supplementary Table 8). *Glyma.19G143300* had sequence polymorphisms between two parents in the upstream, exonic, and UTR regions. *Glyma.19G182400* only showed sequence variation in the intronic region, whereas *Glyma.20g081600* showed sequence variation only in the upstream region. Furthermore, the CDS of above six genes were cloned from the two parents of NJPW-RIL, PI and WH, sequenced and compared. The results showed that only one gene, *Glyma.19G143300*, possessed sequence variations in the CDS region. There are three SNPs in the CDS of *Glyma.19G143300* between the two parental accessions (Fig. 5a), but only one SNP (C to T) at 2258 bp leads to an amino acid change from serine (S) in PI to phenylalanine (F) in WH (Fig. 5b).

*Glyma.19G143300* encodes a leucine-rich repeat receptor-like kinase (LRR-RLK), which has seven tandem copies of leucine-rich repeat (LRR) domains, a transmembrane (TM) domain, and a protein kinase domain (Fig. 5c). The C to T point mutation in the CDS of *Glyma.19G143300* leads to the change of protein kinase domain, from Pkinase_Tyr (tyrosine and serine/threonine protein kinase domain) in PI (Fig. 5c) to STYKc (protein kinase domain with unclassified specificity, with possible dual-specificity of serine-threonine/tyrosine-kinase) in WH (Fig. 5d), which also caused difference in the three-dimensional protein structure between PI and WH (Fig. 5e, f), indicating that this SNP might affect the protein function of *Glyma.19G143300*.

A number of LRR-RLK kinase genes from different species have been found to play roles in controlling seed size, such as *LOC_Os09g12240* (*D61*/*OsBRI1*) (Morinaka et al. 2006) and *LOC_Os04g48760* (*XIAO*) from rice (Jiang et al. 2012), *AT3G19700* (*IKU2*) (Garcia et al. 2003; Luo et al. 2005) and *AT4G39400* (*BRI1*) from Arabidopsis (Jiang et al. 2013), as well as *GRMZM2G149051* (*ZmRLK7*) from maize (He et al. 2020). All of these five proteins have the typical domains of LRR-RLK (Supplementary Fig. 5). A phylogenetic tree was constructed using the full-length protein sequences of above-mentioned LRR-RLK kinases and Glyma.19G143300 (Fig. 5g). It showed that Glyma.19G143300 shared more similarity with the LRR-RLK protein XIAO from rice (Fig. 5g), which has been shown to control seed size (He et al. 2020). These results suggest that *Glyma.19G143300* gene in soybean might also play an important role in controlling seed size/weight as the other known *LRR-RLK* genes.

**Fig. 6:**
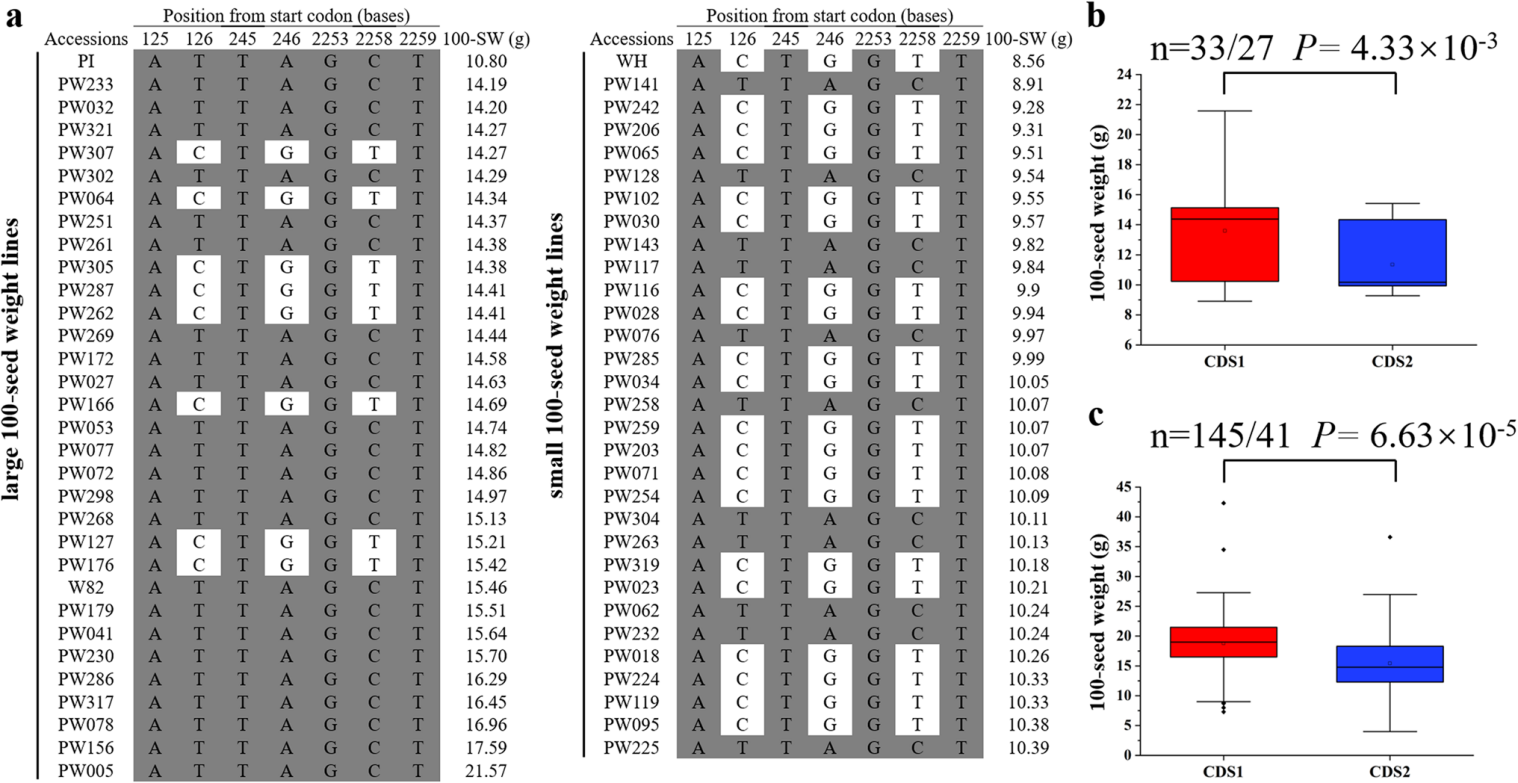
Sequence and allelic variation in *Glyma.19g143300* among soybean recombinant inbred lines (RILs), the two parents and 186 soybean accessions. **a** Sequence variation in the coding region of *Glyma.19g143300* from 60 RILs (with 30 largest and 30 smallest 100-seed weight), the two parental lines of PI and WH, and Williams 82 (W82). The position of the sequence variation is relative to the start codon (ATG), which is shown on the top. 100-SW, 100-seed weight. The RILs were named with PW + number, for example, PW233 represent a RIL derived from the cross of PI × WH. **b** Boxplot of 100-seed weight for two groups of soybean RILs carrying two different CDS types of *Glyma.19g143300*, in the 60 RILs with extreme 100-seed weight. The phenotypic data of 100-seed weight were the mean value across 5 environments. **c** Boxplot of 100-seed weight for two groups of soybean accessions carrying two different CDS types of *Glyma.19g143300*. The sequence variations of *Glyma.19g143300* and phenotypic data of 100-seed weight were downloaded from the database (http://www.mbkbase.org/soybean). Statistical significance of the difference between two groups was determined by two-sided Wilcoxon test. The center bold line represents the median; box edges indicate the upper and lower quantiles; whiskers show the 1.5 × interquartile range and points indicate outliers

In order to verify the relationship between *Glyma.19G143300* polymorphism and 100-seed weight of soybean, the CDS of *Glyma.19G143300* from 30 RILs with extreme large 100-seed weight, 30 RILs with extreme small 100-seed weight, from the NJPW-RIL population, as well as the parents of PI and WH were sequenced and compared. We named the CDS type of *Glyma.19G143300* from the parents of PI and WH as CDS1 and CDS2, respectively. Among the 60 RILs with extreme phenotypes, 33 RILs had CDS1 and 27 RILs showed CDS2 type of *Glyma.19G143300* (Fig. 6a). There was significant difference in average 100-seed weight of soybean RILs between CDS1 and CDS2 groups, which was 13.60 g and 11.34 g, respectively (Fig. 6b). Further, the sequence variations in the CDS of *Glyma.19G143300* were explored using the public available information (http://www.mbkbase.org/). A total of six haplotypes (CDS types) were identified for *Glyma.19G143300* (Supplementary Fig. 6 and Supplementary Table 9) in the database. The CDS1 and CDS2 type of *Glyma.19G143300* corresponds to the CDS type in PI and WH, respectively. The 100-seed weight for 145 soybean accessions containing CDS1 type of *Glyma.19G143300* and 41 accessions with CDS2 type of *Glyma.19G143300* was download from this database. The statistical analysis (Fig. 6c) showed that the CDS1 group had significantly larger average 100-seed weight (18.79 g) than that of the CDS2 group (15.43 g) among the 186 soybean accessions, which is consistent with our result using the 60 RILs with extreme phenotypes (Fig. 6b). These results suggest that CDS1 is the potential superior allele of *Glyma.19G143300* that might improve soybean 100-seed weight compared with CDS2, which needs further verification in future functional studies by transgenic soybean lines.

## Discussion

### Phenotypic variation of 100-seed weight in the soybean NJPW-RIL population

Although great efforts have been made to improve soybean yield to meet the increasing demand (Jeong et al. 2012; Stupar 2010), soybean yield is still low compared with other major crops. Seed weight is an important trait related to yield, and thus, developing soybean cultivars with desirable seed weight is still an important objective for soybean breeding. The 100-seed weight of soybean is a quantitative trait controlled by polygenes (Li et al. 2019b; Yan et al. 2017). Although many QTL associated with 100-seed weight have been identified over the past years, major/stable QTL and candidate genes within these QTL are still desired to be used for soybean breeding program.

In this study, a soybean RIL population, NJPW-RIL, derived from a cross between PI and WH, was used for QTL mapping of 100-seed weight. The 100-seed weight of the NJPW-RIL population was measured under five environments. The ANOVA result revealed that genotype, environment, and genotype × environment interaction had significant effect on the 100-seed weight (Table 2), which is consistent with the previously reported results (Fasoula et al. 2004; Karikari et al. 2019). The heritability in a single environment varied from 83.42% to 97.47%, and the heritability across five environments reached 91.83%, suggesting that the genetic factor makes large contribution to the phenotypic variation in 100-seed weight (Table 1).

The 100-seed weight of the NJPW-RIL population ranged from 8.91 g to 21.57 g based on the average values over five environments, whereas the parents PI and WH had the 100-seed weight of 10.80 g and 8.56 g, respectively, indicating there is a large variation and transgressive segregation in this RIL population (Supplementary Fig. 1a-f). The genetic difference between the two parents, PI (a soybean accession from the USA) and WH (a soybean landrace from China), and their different QTL-allele compositions and recombination, could contribute to the observed variation and transgressive segregation in this RIL population. Among the 38 QTL identified in this study, the alleles with positive effect on 100-seed weight came from both parents, the positive alleles of 32 QTL came from the parent PI (larger seeds), while the positive alleles of the remaining 6 QTL came from WH (smaller seeds) (Supplementary Table 6). The recombination of these alleles leads to the genetic and phenotypic variation in the NJPW-RIL population, and the RILs pyramiding more positive alleles from both parents could lead to larger seed weight than the parent PI, which could be one reason for the observed transgressive segregation in the NJPW-RIL population.

### Major and novel QTL for 100-seed weight of soybean identified in this study

Although a lot of QTL for 100-seed weight have been mapped (https://www.soybase.org), many loci explained a small proportion of the phenotypic variation and mapped to a relatively large genetic/physical interval. The larger population size and higher density of markers would improve the mapping resolution, while enough replications with reduced phenotyping errors, and a high-quality genetic map will improve the accuracy of QTL mapping (Gutierrez-Gonzalez et al. 2011; Zou et al. 2012). In this study, we used a large soybean RIL population consisting of 300 lines and constructed a genetic map of 4702 bin markers using 1.16 million high-quality SNPs genotyped by the whole genome resequencing technology. The average distance between bin markers is 0.74 cM for genetic distance and 200 kb for physical distance, indicating the QTL could be mapped to a smaller region/map interval to achieve a higher mapping resolution. More importantly, the phenotypic data of 100 seed-weight were evaluated under five different environments with three replications within each single environment, which help reducing errors to improve the mapping accuracy.

A total of 38 QTL for 100-seed weight were detected in the soybean NJPW-RIL population, with the average genetic interval of 3.24 cM and the average LOD value of 6.27. Among them, 11 QTL had been mapped to a narrow region (genetic interval < 2 cM), which would help us to further fine map the QTL and identify the candidate genes to improve the accuracy of marker-assisted selection in soybean breeding program. Four major QTL, including *qSw-19-1*, *qSw-19-5*, *qSw-20-2*, and *qSw-20-3*, had a large contribution to the phenotypic variation (*R*^2^ ≥ 10% for each QTL). Four QTL, *qSw-7-1*, *qSw-10-1*, *qSw-14-1* and *qSw-16-1*, could be novel, while 34 QTL overlapped with the previously reported QTL in Soybase database (https://www.soybase.org), by comparing their physical locations (Supplementary Table 6). And 12 QTL were identified in multiple environments (≥ 2). Out of these 12 stable QTL, three QTL, including *qSw-19-5*, *qSw-20-2*, and *qSw-20-3*, explained a large phenotypic variation (*R*^2^ ≥ 10%) and thus, were considered as the major and stable QTL (Supplementary Table 6). The first major QTL *qSw-19-1* was detected in the 2015JP environment, which overlaps with the previously reported QTL *Seed weight 35-7* in Soybase (Han et al. 2012). The second major QTL *qSw-19-5* can be detected in three environments and by the mean values across five environments (MEAN), which overlaps with the previously mapped QTL of *Seed weight 7-7* (Orf et al. 1999), *Seed weight 17-1* (Stombaugh et al. 2004), and *Seed weight 43-4* (Kuroda et al. 2013). The third major QTL *qSw-20-2* could be identified in four environments and MEAN, and overlaps with the QTL of *Seed weight 8-1* (Sebolt et al. 2000), *Seed weight 34-5* and *Seed weight 35-5* (Han et al. 2012). The fourth major QTL *qSw-20-3* was detected in four environments and MEAN, which overlaps with the QTL *Seed weight 9-1* (Sebolt et al. 2000). The overlapping of QTL identified in this study with the published QTL for soybean seed weight suggests the accuracy of these QTL.

### Comparisons between QTL mapping using whole genome sequencing and SNP arrays

Recent advances in high-throughput genotyping technologies have facilitated studies on crop genetics and breeding, which mainly includes SNP array and whole genome sequencing. SNP array is an affordable, efficient, and robust method for high-throughput SNP genotyping. In soybean, several SNP arrays with different marker densities (3 K, 6 K, 50 K, 180 K, 355 K, 618 K) have been developed (Akond et al. 2013; Lee et al. 2015; Li et al. 2022b; Patil et al. 2018; Song et al. 2013; Wang et al. 2016), and SoySNP50K BeadChip and SoySNP6K (Akond et al. 2013; Song et al. 2013) had been widely used for soybean QTL mapping (Assefa et al. 2019; Diers et al. 2018). However, the main drawbacks of common SNP arrays include the inflexibility with fixed number of SNPs on the chips (which could not discover new SNPs), and the ascertainment bias (such as eliminating SNPs with low minor allele frequency) depending on the population used in the SNP discovery panel (Geibel et al. 2021). Whole genome sequencing can detect SNPs at whole genome level without pre-selection to avoid ascertainment bias, and discover more genetic variation. However, whole genome sequencing requires well trained scientists, more computational resources and time to analyze the data. With the rapid decrease in sequencing cost, whole genome sequencing is more suitable for high-density marker genotyping, whereas SNP array is a cost-efficient and rapid genotyping method for low to moderate density SNP markers, especially for larger sample size. The most recent liquid chip technology is a new high-throughput genotyping platform using genotyping by target sequencing (GBTS) technology, which has been successfully developed in several crops such as soybean (Liu et al. 2022) and maize (Ma et al. 2022), and would be another useful tool for crop genetic study and breeding.

In this study, a total of 1,161,784 SNPs that showed polymorphism between the parents of soybean NJPW-RIL were obtained by whole genome sequencing, which provides a new set of markers complementary to the 52,041 SNPs on the SoySNP50K BeadChip (Song et al. 2013) and 5,376 SNPs on the SoySNP6K BeadChip (Akond et al. 2013). In terms of identified QTL numbers, we detected more QTL for seed weight than the other study on QTL mapping of seed weight using SoySNP50K BeadChip (Assefa et al. 2019). A total of 38 QTL for 100-seed weight were detected using the genetic map containing 4702 bin markers in this study, whereas 14 significant SNPs associated with seed weight were identified in a genome-wide association study on 419 diverse soybean accessions genotyped by the SoySNP50K BeadChip (Assefa et al. 2019). Moreover, the average physical distance between bin markers on the genetic map established in this study is 200 kb, whereas the average linkage disequilibrium decay distances for euchromatin and heterochromatin regions were estimated as 238 kb and 1,648 kb in the 419 soybean accessions, respectively (Assefa et al. 2019). These suggest that this study provides additional discovery power to detect more SNPs and QTLs and helps to narrow down the QTL intervals comparing with the study using SoySNP50K BeadChip (Assefa et al. 2019).

### Candidate gene prediction for 100-seed weight in soybean

Several categories of genes have been found to play important roles in regulating seed size/weight, including ubiquitin–proteasome pathway, G-protein signaling, MAPK signaling, phytohormones, and transcriptional regulatory factors (Li et al. 2019a). The ubiquitin–proteasome pathway related genes, such as *DA1* (Li et al. 2008), *DA2* (Xia et al. 2013), *PUB25* and *PUB26* (Li et al. 2021) from Arabidopsis, regulate seed and organ size by restricting the period of cell proliferation. OsRac1, a ROP GTPases protein, modulates rice grain size by promoting cell division (Zhang et al. 2019). OsMKK4 and OsMAPK6, the mitogen-activated protein kinases, are positively associated with grain size in rice (Duan et al. 2014; Liu et al. 2015). The hormone-related genes, including *AUXIN RESPONSE FACTOR 2* gene (*ARF2*) from Arabidopsis (Schruff et al. 2006), gibberellin-related gene *GA20OX* from soybean and Arabidopsis (Lu et al. 2016; Plackett et al. 2012), brassinolide-related gene *BZR1* and/or *BES1*/*BZR2* and *PP2C-1* from Arabidopsis and soybean (Jiang et al. 2015, 2013; Lu et al. 2017), have been reported to regulate seed weight/size. Several transcriptional regulatory factor genes have been identified as important regulators of seed size in plants, including transcription factor genes such as *SoyWRKY15* from soybean (Gu et al. 2017), and *BS1* from *Medicago* and soybean (Ge et al. 2016).

In the present study, we tried to identify the candidate genes within the physical regions of four major QTL for 100-seed weight in soybean. The RNA-seq data of the annotated genes within these four major QTL showed that 34 genes had higher expression levels in seeds than other soybean tissues (Supplementary Fig. 4 and Supplementary Table 7). As mentioned above, it has been known that ubiquitin–proteasome pathway, G-protein signaling, MAPK signaling, phytohormones, and transcriptional regulatory factors play important roles in seed development (Li and Li 2016; Li et al. 2019a). Therefore, six out of 34 genes with the above annotations were identified as candidate genes for 100-seed weight in this study. Among these six candidate genes, five of them, including *Glyma.19G143300*, *Glyma.19G182400*, *Glyma.20G053200*, *Glyma.20G055900*, and *Glyma.20G062700*, showed higher relative expression levels in the seeds of the parental accession PI (larger seeds) than the other parental accession WH (smaller seeds) at different seed developmental stages (Fig. 4). Further sequence variation analyses suggest that *Glyma.19G143300*, a gene encoding an LRR-RLK kinase, is the most likely candidate gene for soybean 100-seed weight. A SNP (C to T) in the coding region of *Glyma.19G143300* leads to an amino acid change from serine to phenylalanine in its protein, and different predicted protein structures between PI and WH. The predicted protein has a Pkinase_Tyr (tyrosine and serine/threonine protein kinase domain) in PI, while it contains a STYKc (protein kinase domain with unclassified specificity, with possible dual-specificity of serine-threonine/tyrosine-kinase) in WH at the C terminal (Fig. 5). How would the change of C-terminal domain affect the function of protein and thus leading to the phenotypic changes in 100-seed weight needs further investigation in future study.

LRR kinases have been known as one of the typical regulators to control seed size/weight (Li et al. 2019a). In Rice, *D61*/*OsBRI1,* which belongs to the LRR-RLK family, plays an important role in regulation of the rice grain size by affecting cell expansion (Morinaka et al. 2006). LRR kinases participate in diverse signaling pathways to regulate cellular processes. *XIAO* encodes an LRR kinase that regulates the signaling and homeostasis of brassinosteroids and cell cycling to control organ size in rice (Jiang et al. 2012). *IKU2*, a LRR kinase gene, controls seed size in Arabidopsis (Garcia et al. 2003; Luo et al. 2005). *ZmRLK7* encodes a putative LRR-RLK in maize, and overexpression of *ZmRLK7* increased the organ size and seed weight. ZmRLK7 restricts both cell expansion and proliferation to play key roles in regulating the petal size in maize (He et al. 2020). These results suggested that LRR-RLK kinases play important roles in regulating seed size/weight in plant species. *Glyma.19G143300* also encodes an LRR-RLK kinase and shared conserved/typical domains with the proteins mentioned above (Supplementary Fig. 5), suggesting that *Glyma.19G143300* could also have the potential role in regulating the seed size/weight in soybean as the other LRR-RLK members. Further study is needed for its functional validation.

The relationship between the sequence variation of *Glyma.19G143300* and 100-seed weight was analyzed in a subset of 60 NJPW-RILs with extreme phenotypes, including 30 RILs with largest 100-seed weight and 30 RILs with smallest 100-seed weight in the RIL population. The results showed that there were 33 lines have CDS1 type of *Glyma.19G143300* while 27 lines contain CDS2 type of *Glyma.19G143300*, and significant difference in 100-seed weight was observed between the two groups of CDS1 and CDS2 (Fig. 6). Most (22/30 = 73.33%) lines with large 100-seed weight belong to CDS1 group, while 63.33% (19/30) of lines with small 100-seed weight have CDS2 type of *Glyma.19G143300*. These results suggested that although *Glyma.19G143300* within the major QTL explained 11.60% of the phenotypic variation for 100-seed weight in the NJPW-RIL population, there are other loci controlled 100-seed weight as well.

Among the candidate genes within the four major QTL regions for 100-seed weight, in addition to *Glyma.19G143300*, the other five genes with differential expression levels between the two parents could also be candidate genes. We compared the resequencing data of the two parental lines PI and WH and found that two genes, including *Glyma.19G143300* and *Glyma.20g081600*, had sequence polymorphism in the 2.0-kb promoter regions between the two parents (Supplementary Table 8), which could result in their differential expression levels between the two parents. Their roles in regulation of soybean seed weight should be investigated in follow up studies.

The candidate gene mining criteria used in the study, including preferential expression in seeds, homologous genes in regulating seed size in other species, differential expression patterns, non-synonymous SNPs within the candidate genes, could provide us a good indication for the function of the candidate genes and help us to quickly identify some candidate genes. However, it is possible that the causal genes within the major QTLs were missed out using above criteria. For example, the differential expression of a gene might be caused by the variation of its trans-acting regulatory gene underlying a different QTL locus. Also, genes underlying seed trait QTLs do not have to express at a high level or show differential expression in seeds. The newly identified gene *POWR1*, which pleiotropically regulates soybean seed quality and yield, is preferentially expressed in seed coat and flowers and did not show high expression level or significant differential expression between two alleles in seeds (Goettel et al. 2022). Thus, some genes with moderate expression levels in seeds or with new function in regulating seed weight might be missed in the candidate list. However, through screening highly expressed genes in seeds, some important genes related to seed traits have been identified, such as *GmSWEET39* (Miao et al. 2020), *GmWRKY15a* (Gu et al. 2017), *GmGA20OX* and *GmNFYA* (Lu et al. 2016).

## Supplementary Information

Below is the link to the electronic supplementary material.

Supplementary file 1 (PDF 2088 kb)

## Data Availability

The datasets in the current study are available in the supplementary information published online or from the corresponding author on reasonable request. The raw sequencing data from this study have been deposited in the Genome Sequence Archive in BIG Data Center (https://bigd.big.ac.cn/), Beijing Institute of Genomics (BIG), Chinese Academy of Sciences, under the accession number: PRJCA013517.
