# Supplementary material for: Identification of major quantitative trait loci and candidate genes for seed weight in soybean
Source: Theor Appl Genet. 2023 Jan 23;136(1):22. doi: 10.1007/s00122-023-04299-w (PMC9870841; doi:10.1007/s00122-023-04299-w)
Supplement: Supplementary file 1 — (PDF 2088 kb) [file 122_2023_4299_MOESM1_ESM.pdf]

Theoretical and Applied Genetics

Supplementary material

Identification of major quantitative trait loci and candidate genes for seed weight in soybean

Mengge Xu · Keke Kong · Long Miao · Jianbo He · Tengfei Liu · Kai Zhang · Xiuli Yue · Ting Jin · Junyi Gai · Yan Li*

National Key Laboratory of Crop Genetics and Germplasm Enhancement, National Center for Soybean Improvement, Key Laboratory for Biology and Genetic Improvement of Soybean (General, Ministry of Agriculture), Jiangsu Collaborative Innovation Center for Modern Crop Production, Nanjing Agricultural University, Nanjing, China

*To whom correspondence should be addressed.
E-mail: yanli1@njau.edu.cn

Supplementary information

Supplementary Fig. 1 Distribution of 100-seed weight in the NJPW-RIL population.

Supplementary Fig. 2 Statistics of mapping rate of the NJPW-RIL population.

Supplementary Fig. 3 Distribution of QTL for 100-seed weight on soybean chromosomes identified in the NJPW-RIL population.

Supplementary Fig. 4 Expression analyses of annotated genes in the four major 100-seed weight QTL genomic regions.

Supplementary Fig. 5 Sequence alignment of Glyma.19G143300 and the known leucine-rich repeat receptor-like kinase (LRR-RLK) proteins.

Supplementary Fig. 6 Sequence and allelic variation in the CDS of *Glyma.19G143300* among different soybean accessions.

Supplementary Table 1 Primers used in this study.

Supplementary Table 2 The 100-seed weight of 300 lines of the NJPW-RIL population in five environments.

Supplementary Table 3 Summary of the sequencing data for two parents.

Supplementary Table 4 Summary of mapping rates for two parents.

Supplementary Table 5 Statistics of the marker information of the genetic linkage map for the NJPW-RIL population.

Supplementary Table 6 The quantitative trait loci (QTL) for 100-seed weight identified in the NJPW-RIL population under multiple environments.

Supplementary Table 7 Thirty-four possible candidate genes and annotations related to 100-seed weight.

Supplementary Table 8 Sequence variation of six candidate genes in two parental lines based on the re-sequencing data.

Supplementary Table 9 The 100-seed weight of soybean accessions carrying two different CDS types of *Glyma.19G143300*.

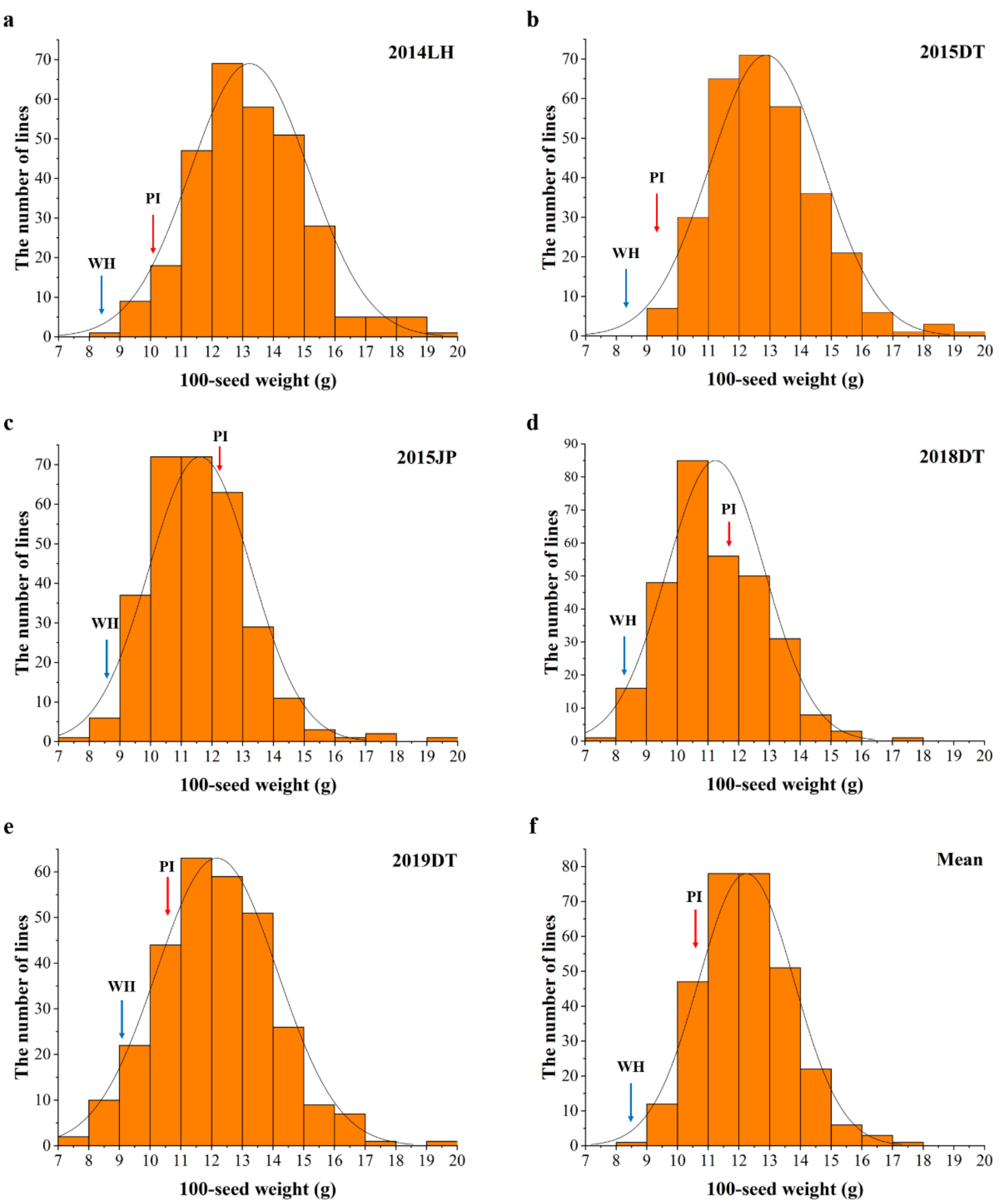

Supplementary Fig. 1 Distribution of 100-seed weight in the NJPW-RIL population. a, **b**, **c**, **d** and **e** represent the histogram of the 100-seed weight in 2014LH, 2015DT, 2015JP, 2018DT and 2019DT, respectively. 2014LH, experiment at Liuhe in 2014; 2015DT, experiment at Dangtu in 2015; 2015JP, experiment at Jiangpu in 2015; 2018DT, experiment at Dangtu in 2018; 2019DT, experiment at Dangtu in 2019. Data represents the average value of three replications under each environment. **f** Histogram of the mean values of 100-seed weight across five environments of 2014LH, 2015DT, 2015JP, 2018DT and 2019DT. Arrows indicate the 100-seed weight of the two parental lines in that year.

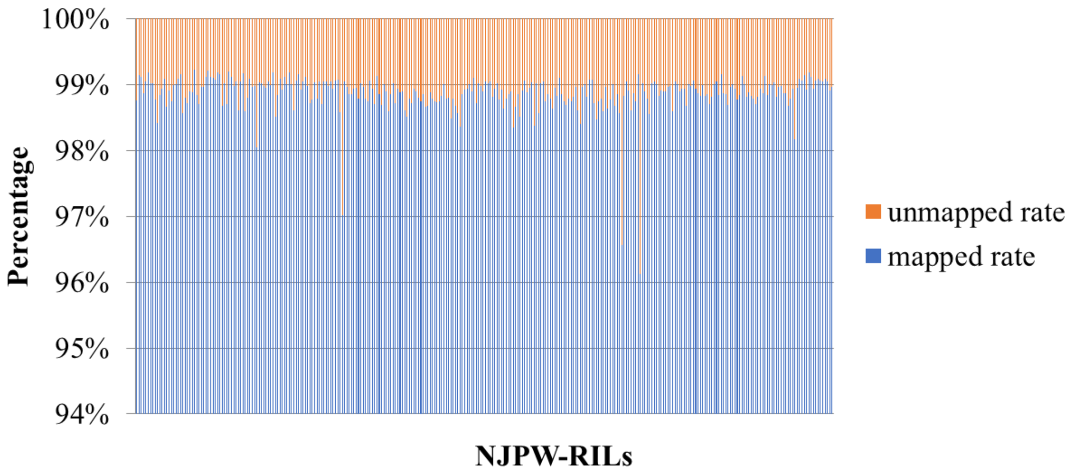

Supplementary Fig. 2 Statistics of mapping rate of the NJPW-RIL population. In total, 300 NJPW-RILs were sequenced with an average of 2× depth. And nearly 81.89% bases (bp) could be uniquely mapped to the Williams 82 reference genome.

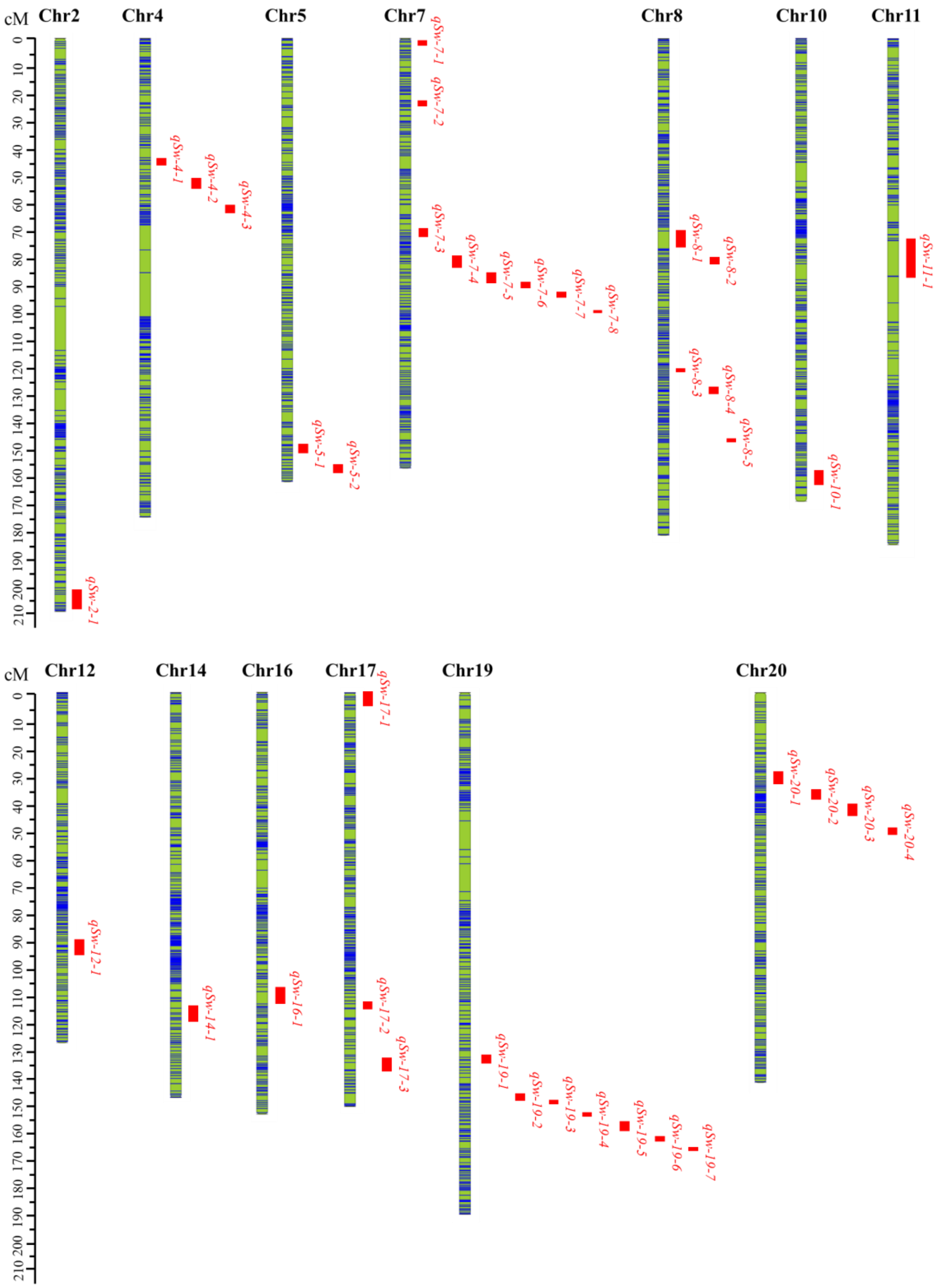

Supplementary Fig. 3 Distribution of QTL for 100-seed weight on soybean chromosomes identified in the NJPW-RIL population. The vertical green bars represent the soybean chromosomes, and horizontal blue lines represent the bin markers. All QTL of 100-seed weight were detected by CIM model. The red boxes represent the 1-LOD interval (cM) of the identified QTL. The ruler on the left is shown in the unit of cM.

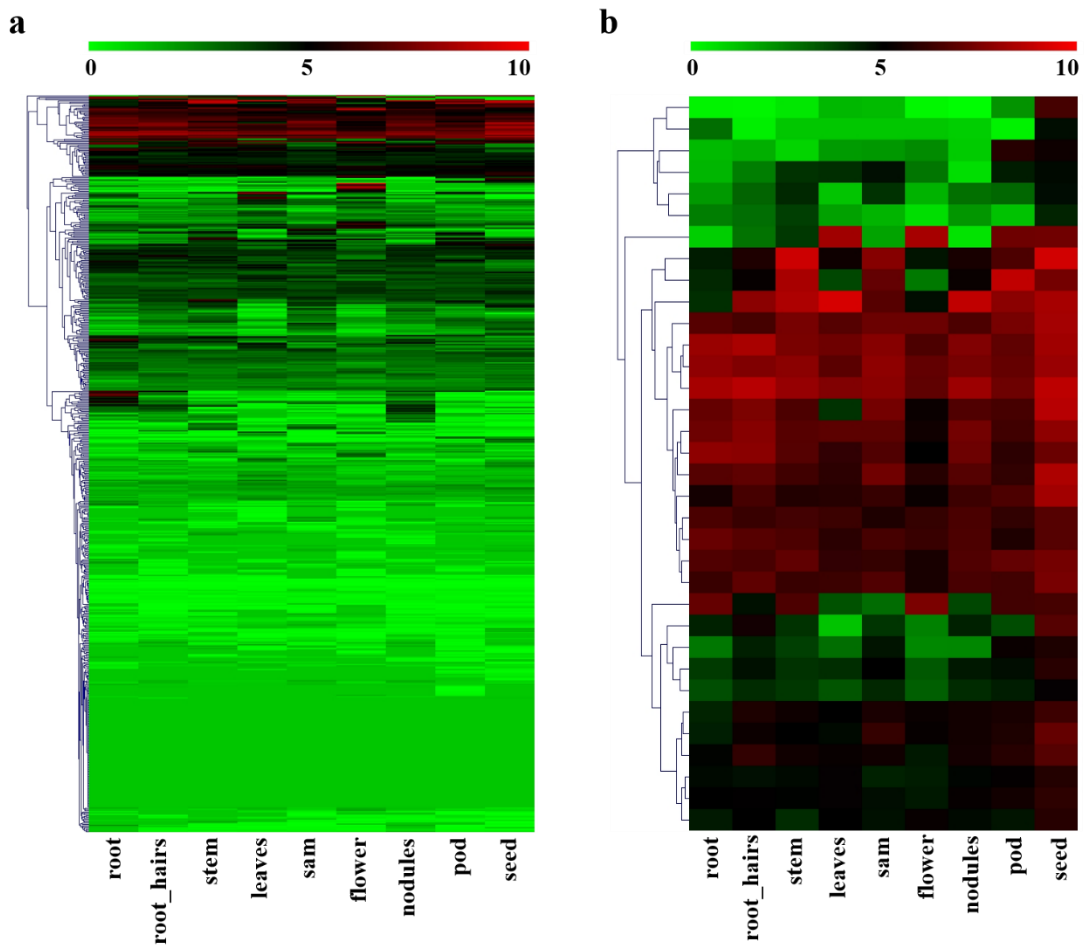

Supplementary Fig. 4 Expression analyses of annotated genes in the four major 100-seed weight QTL genomic regions. a Heatmap of 596 annotated genes within the four major QTL, *qSw-19-1*, *qSw-19-5*, *qSw-20-2* and *qSw-20-3*, in different soybean (*Glycine max*) tissues based on RNA-seq data. **b** Heatmap of 34 candidate genes which have higher expression levels in soybean seeds than other tissues ($\Delta FPKM = FPKM_{seed} - FPKM_{mean} \geq 10$). The raw reads from Phytozome (https://phytozome-next.jgi.doe.gov/) were transformed to $\log_2(FPKM + 1)$. FPKM, fragments per kb of transcript per million mapped reads.

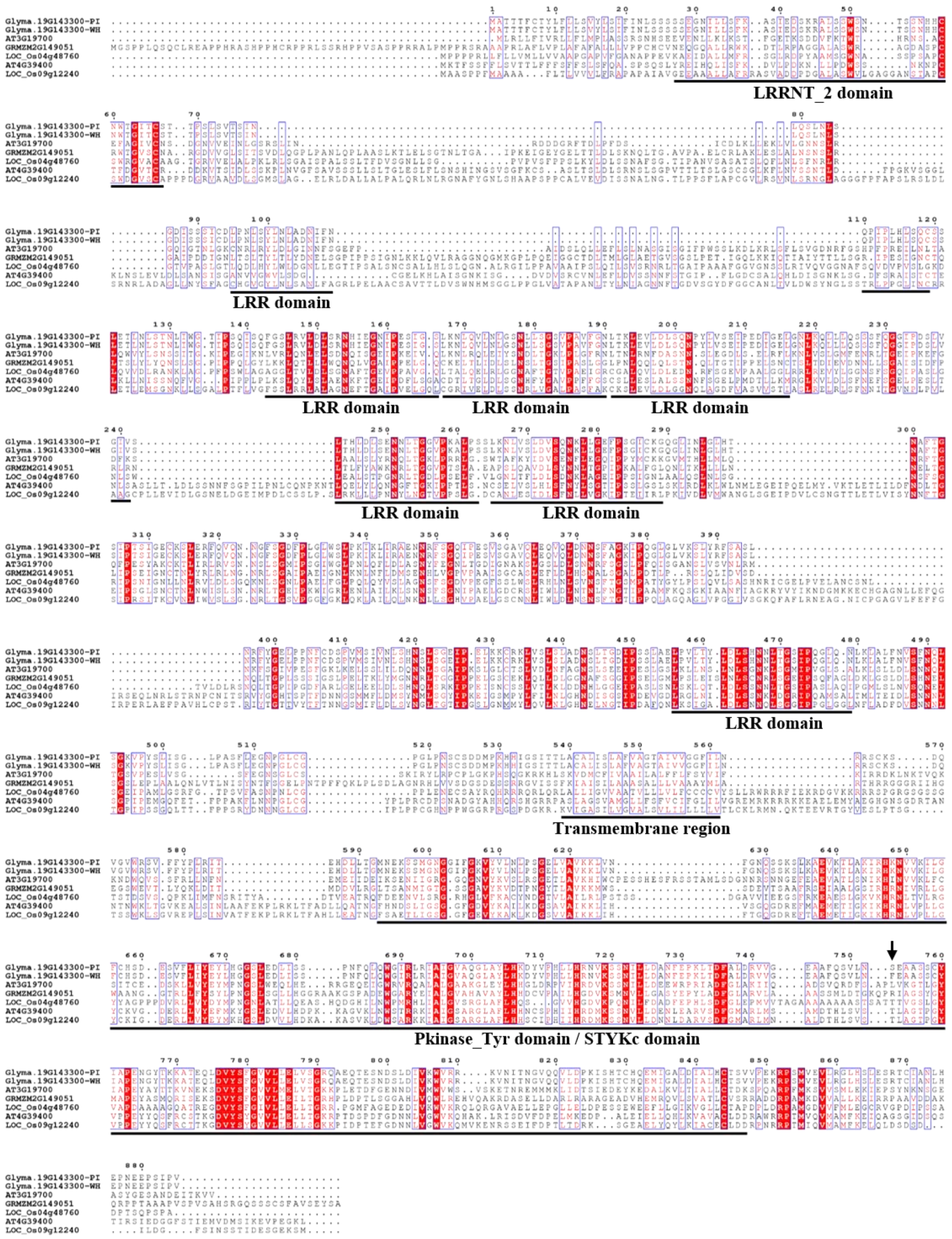

Supplementary Fig. 5 Sequence alignment of Glyma.19G143300 and the known leucine-rich repeat receptor-like kinase (LRR-RLK) proteins. Amino acid sequence alignment of Glyma.19G143300 (soybean) and the known LRR-RLK proteins in Arabidopsis, maize, and rice. The black lines indicate the conserved/typical domains among different species. The amino acid change due to a point mutation between PI and WH is indicated by the black arrow.

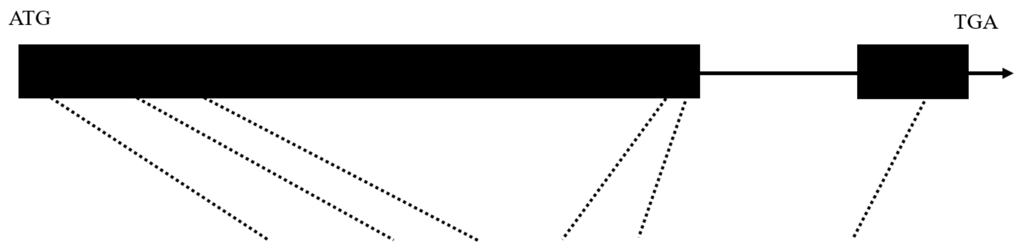

Haplotype (CDS type)	+42	+126	+246	+2243	+2258	+2391
T1 (CDS1)	T	T	A	A	C	G
T2 (CDS2)	T	C	G	A	T	G
T3 (CDS3)	T	C	G	A	C	G
T4 (CDS4)	T	C	G	A	C	A
T5 (CDS5)	T	C	G	T	C	G
T6 (CDS6)	C	T	A	A	C	G

Supplementary Fig. 6 Sequence and allelic variation in the CDS of *Glyma.19G143300* among different soybean accessions. T1-T6 (CDS1-CDS6) represents six different haplotypes (CDS types) of *Glyma.19G143300*. The position of start codon is considered as + 1. The black boxes represent exons and the lines between boxes represent intron. Nucleotide polymorphisms are displayed at their corresponding positions. The reference genome is Williams 82 (*Glycine maxv2.1* genome).

Supplementary Table 1 Primers used in this study.

Name	Primer sequence (5' to 3')	Note
Glyma.19G143300-qRT-F	CAAAGGCTCTACCGTCTTCTCT	qRT-PCR
Glyma.19G143300-qRT-R	AGGCATTTGTGTGGAGACCG	qRT-PCR
Glyma.19G182400-qRT-F	GGACAAGCACAAGCCTATGTATC	qRT-PCR
Glyma.19G182400-qRT-R	TTGTTGCACCAGGAACTTGGA	qRT-PCR
Glyma.20G053200-qRT-F	TTTGAGCGGGACATTGGGTT	qRT-PCR
Glyma.20G053200-qRT-R	CGTCCATTCCACCAAGGTCA	qRT-PCR
Glyma.20G055900-qRT-F	CACTCACCCAGGATTCCTCCC	qRT-PCR
Glyma.20G055900-qRT-R	GGGCAGGTTTGACCTCAGT	qRT-PCR
Glyma.20G062700-qRT-F	TCTCCCAAGCCTCCTCCAAAAA	qRT-PCR
Glyma.20G062700-qRT-R	GGAGATGACTTGGGTGGTGGA	qRT-PCR
Glyma.20G081600-qRT-F	GGACTTGTTCGGAGAGATGGA	qRT-PCR
Glyma.20G081600-qRT-R	AGAGTCATCGAAATCGTCTTGG	qRT-PCR
GmUKN1-qRT-F	GCGAATGTTACGGAGGCTCTG	qRT-PCR
GmUKN1-qRT-R	ATCTGAATGGTGGCGGGATCAA	qRT-PCR
Glyma.19G143300-F	CCCACACACTAAGCACTGATA	cloning *Glyma.19G143300* CDS for sequencing
Glyma.19G143300-R	TCAGGAGTACCATCACGTATTTGT	cloning *Glyma.19G143300* CDS for sequencing
Glyma.19G182400-F	TGACTCGGTGGAGACTTGAG	cloning *Glyma.19G182400* CDS for sequencing
Glyma.19G182400-R	TCAATGGGCTTCTCCTGAACC	cloning *Glyma.19G182400* CDS for sequencing
Glyma.20G053200-F	TCACAGTCCCATCCCTGCTC	cloning *Glyma.20G053200* CDS for sequencing
Glyma.20G053200-R	CATCAGCTACAATCCAGGGCA	cloning *Glyma.20G053200* CDS for sequencing
Glyma.20G055900-F	GTGCACCTAGCATATCCCAAAA	cloning *Glyma.20G055900* CDS for sequencing
Glyma.20G055900-R	AGCCTTACCTACAAGAGCAGAAA	cloning *Glyma.20G055900* CDS for sequencing
Glyma.20G062700-F	AAAGGCCCCCTCATTGTTTCT	cloning *Glyma.20G062700* CDS for sequencing
Glyma.20G062700-R	CAATTCCTTGGTCCTTGGGGA	cloning *Glyma.20G062700* CDS for sequencing
Glyma.20G081600-F	TCCTTCGGAGGAATGCACAG	cloning *Glyma.20G081600* CDS for sequencing
Glyma.20G081600-R	ACCGAAACCCTAACCGAGAAA	cloning *Glyma.20G081600* CDS for sequencing

Supplementary Table 2 The 100-seed weight of 300 lines of the NJPW-RIL population in five environments.

Line	100-seed weight (g) ± Standard deviation					
	2014LH	2015DT	2015JP	2018DT	2019DT	Mean
NJAU_PW001	14.37±0.28	12.2±0.68	12.49±0.51	13.14±0.42	13.58±1.22	13.21±1.01
NJAU_PW002	13.71±0.25	13.52±0.03	13.19±0.44	11.98±0.30	13.38±0.98	13.13±0.81
NJAU_PW003	15.55±1.07	14.83±0.09	13.48±1.32	11.97±1.43	11.64±2.77	13.49±2.13
NJAU_PW004	13.12±0.40	14.71±1.04	12.25±0.24	11.81±0.73	11.46±0.85	12.70±1.39
NJAU_PW005	23.38±1.26	24.43±1.78	20.06±2.32	.	17.91±1.50	21.57±3.14
NJAU_PW006	10.09±2.80	11.31±1.59	10.30±1.12	10.93±0.36	12.44±0.63	11.02±1.63
NJAU_PW007	11.90±0.62	12.34±0.58	12.21±0.81	10.49±1.09	10.99±1.04	11.54±1.04
NJAU_PW008	12.47±0.11	11.52±0.66	9.76±1.08	10.14±0.64	13.23±0.25	11.65±1.41
NJAU_PW010	10.40±0.38	11.25±0.24	10.60±0.71	9.86±1.04	10.16±0.39	10.45±0.72
NJAU_PW011	13.39±0.89	12.47±1.06	12.32±0.36	11.34±0.67	9.63±0.66	11.83±1.47
NJAU_PW012	14.38±0.40	13.39±0.21	12.01±1.08	13.03±1.88	14.26±1.48	13.35±1.37
NJAU_PW013	12.34±0.48	11.77±0.92	9.72±0.84	10.00±1.54	11.11±1.44	10.99±1.40
NJAU_PW015	13.52±0.29	13.52±0.92	12.04±0.60	12.67±0.37	10.80±0.46	12.51±1.16
NJAU_PW016	12.21±0.32	11.31±0.91	10.86±0.70	9.77±0.48	10.80±2.86	11.00±1.27
NJAU_PW017	11.32±0.55	13.30±1.55	10.72±0.79	8.02±0.23	9.72±0.41	10.80±1.87
NJAU_PW018	10.26±0.98	11.16±0.53	8.73±0.40	9.25±0.74	11.38±0.38	10.26±1.18
NJAU_PW019	9.10±1.20	12.29±1.72	11.83±0.45	10.02±0.71	10.59±1.38	10.77±1.57
NJAU_PW020	11.89±0.13	12.12±0.37	10.41±0.96	8.78±0.65	12.05±1.51	11.05±1.53
NJAU_PW021	11.53±0.08	11.82±0.87	10.63±0.18	9.33±0.25	10.02±0.51	10.66±1.04
NJAU_PW022	12.34±1.21	12.08±2.28	11.54±0.25	10.28±1.19	11.83±0.54	11.62±1.37
NJAU_PW023	10.66±1.37	10.54±0.58	9.87±1.55	10.31±2.39	9.68±3.00	10.21±1.71
NJAU_PW024	15.39±0.80	12.76±0.10	11.33±0.44	11.75±1.80	13.64±1.23	12.99±1.81
NJAU_PW025	11.67±0.26	11.33±0.90	10.38±0.77	10.37±1.07	10.31±1.22	10.81±0.97
NJAU_PW026	14.52±1.12	12.50±0.61	12.60±0.41	12.32±2.26	12.87±3.33	12.96±1.80
NJAU_PW027	15.36±0.74	15.82±1.15	14.37±0.91	13.65±2.69	13.93±1.12	14.63±1.53
NJAU_PW028	9.68±0.16	10.78±0.43	9.92±0.45	10.20±1.15	9.09±0.43	9.94±0.78
NJAU_PW029	12.13±1.82	10.57±0.65	10.30±0.32	9.08±1.64	11.25±0.90	10.67±1.46
NJAU_PW030	9.74±0.31	10.31±0.33	9.95±0.45	8.25±0.37	9.60±0.55	9.57±0.80
NJAU_PW031	13.10±0.18	12.82±0.63	11.46±0.01	11.43±0.78	13.03±1.36	12.43±1.02
NJAU_PW032	14.97±0.93	13.99±0.79	12.81±0.36	13.73±1.00	15.02±0.6	14.20±1.05
NJAU_PW033	14.79±0.47	15.46±1.30	14.39±1.12	12.72±0.27	13.32±1.89	14.14±1.42
NJAU_PW034	11.08±0.15	10.25±0.24	8.65±0.77	10.35±0.48	9.94±0.28	10.05±0.90
NJAU_PW035	15.98±0.34	14.28±2.11	13.23±0.99	11.66±0.99	10.69±0.48	13.17±2.18
NJAU_PW036	14.74±0.79	14.35±1.00	11.24±1.92	11.37±1.36	13.55±0.87	13.18±1.78
NJAU_PW037	13.58±0.25	11.82±0.78	11.87±1.20	11.74±0.48	9.57±0.34	11.72±1.44
NJAU_PW040	12.88±0.75	12.10±0.31	10.21±0.83	9.95±0.49	11.56±0.09	11.42±1.23
NJAU_PW041	17.60±0.36	14.88±0.59	13.97±2.17	15.80±1.60	16.61±1.55	15.64±1.77
NJAU_PW042	13.27±0.91	14.74±1.93	12.48±0.75	13.49±1.82	14.39±0.22	13.68±1.34
NJAU_PW043	12.37±0.82	10.20±0.89	9.77±0.33	10.94±1.13	12.59±0.61	11.28±1.34

Line	100-seed weight (g) ± Standard deviation					
	2014LH	2015DT	2015JP	2018DT	2019DT	Mean
NJAU_PW044	14.52±0.79	13.90±2.67	12.00±1.27	12.88±0.88	12.51±0.59	13.24±1.54
NJAU_PW045	12.82±0.83	12.94±0.57	11.18±0.74	11.06±0.09	12.79±1.38	12.32±1.11
NJAU_PW046	12.06±0.34	11.20±0.39	11.25±0.42	10.04±2.12	8.46±0.56	10.6±1.56
NJAU_PW047	14.71±0.22	12.12±0.26	10.74±0.58	12.83±2.15	11.26±1.05	12.33±1.72
NJAU_PW048	13.58±0.61	13.25±0.63	11.14±0.88	8.99±0.63	12.47±1.82	11.89±1.94
NJAU_PW049	12.65±0.41	12.93±0.51	10.95±0.33	11.26±1.58	15.62±1.43	12.80±1.92
NJAU_PW050	14.06±0.83	13.22±1.22	10.55±0.61	12.36±1.45	13.04±1.05	12.80±1.45
NJAU_PW052	12.05±0.78	12.45±0.03	11.83±0.03	11.35±1.24	12.98±1.61	12.21±0.99
NJAU_PW053	16.36±0.87	16.29±0.31	13.07±1.22	14.07±0.49	13.92±3.01	14.74±1.89
NJAU_PW054	11.22±0.54	11.77±1.26	10.79±1.64	9.49±0.11	12.19±1.70	11.09±1.41
NJAU_PW055	13.11±0.42	13.99±0.59	11.96±0.93	10.48±0.49	13.04±2.70	12.52±1.68
NJAU_PW056	11.06±0.21	12.71±2.67	12.11±0.63	12.46±1.14	11.68±0.65	12.07±1.32
NJAU_PW057	13.48±0.13	13.37±0.70	11.73±0.84	10.77±0.91	12.24±2.03	12.32±1.41
NJAU_PW058	12.17±0.77	11.10±0.66	10.86±0.02	9.69±0.29	11.65±2.68	11.07±1.28
NJAU_PW059	12.63±1.15	10.68±0.43	9.44±1.28	12.12±1.71	11.18±1.38	11.21±1.57
NJAU_PW061	10.73±0.76	11.00±0.33	10.57±0.30	10.46±0.39	10.65±0.61	10.66±0.47
NJAU_PW062	11.61±0.73	10.10±0.61	9.71±0.69	9.26±0.57	10.52±1.03	10.24±1.04
NJAU_PW063	11.45±0.87	12.74±1.80	11.65±0.54	11.70±0.95	12.43±1.46	12.00±1.15
NJAU_PW064	15.25±0.71	15.17±0.12	14.84±0.21	12.68±1.34	13.95±2.37	14.34±1.51
NJAU_PW065	11.05±1.28	9.90±0.57	9.00±0.83	8.77±0.39	8.11±1.61	9.51±1.34
NJAU_PW066	12.8±0.17	11.65±0.36	10.00±1.01	9.56±0.15	11.50±1.00	11.1±1.34
NJAU_PW067	11.27±0.11	11.38±1.59	11.09±1.78	9.42±0.21	11.18±1.31	10.87±1.28
NJAU_PW068	12.92±0.70	13.22±1.08	12.86±0.42	11.31±0.34	12.49±1.34	12.54±1.03
NJAU_PW069	13.23±0.09	11.33±1.21	12.46±1.27	10.11±0.60	16.42±0.00	12.20±1.94
NJAU_PW070	12.40±0.52	12.33±0.80	11.72±0.74	10.87±0.40	12.16±0.57	11.90±0.79
NJAU_PW071	9.98±1.08	11.04±0.13	10.67±0.43	8.94±0.89	9.77±0.59	10.08±0.97
NJAU_PW072	13.83±0.49	13.68±0.74	13.22±0.23	12.73±1.08	20.30±0.97	14.86±3.05
NJAU_PW073	12.99±1.47	13.17±0.95	13.06±0.09	12.2±0.50	13.64±1.95	13.01±1.16
NJAU_PW074	13.25±0.15	11.96±0.17	11.05±0.55	10.85±2.37	14.86±0.12	12.49±1.83
NJAU_PW075	12.39±0.20	12.07±0.13	10.87±0.14	10.06±0.94	13.48±1.39	11.77±1.39
NJAU_PW076	9.66±1.94	11.73±1.14	9.81±0.49	9.08±1.01	9.27±0.76	9.97±1.40
NJAU_PW077	15.93±0.35	16.27±0.25	14.84±0.76	14.8±0.54	12.77±1.20	14.82±1.40
NJAU_PW078	18.92±2.59	18.46±0.40	17.88±1.35	12.91±0.60	.	16.96±2.92
NJAU_PW079	12.67±0.20	12.86±0.56	12.48±0.60	9.17±0.39	11.69±1.05	11.77±1.51
NJAU_PW080	13.14±0.47	13.86±1.02	12.02±0.49	11.15±0.22	12.39±1.10	12.52±1.15
NJAU_PW081	12.30±0.84	9.59±1.22	9.91±0.62	12.49±0.81	11.49±0.52	11.15±1.43
NJAU_PW083	12.12±0.93	10.02±1.06	9.62±0.47	9.89±0.99	11.08±2.38	10.55±1.48
NJAU_PW084	15.13±0.64	14.23±0.65	12.23±0.78	12.14±0.16	10.95±0.63	12.98±1.69
NJAU_PW085	14.55±0.52	12.73±0.93	13.61±0.71	10.85±0.82	11.42±3.19	12.63±1.94
NJAU_PW086	12.83±0.17	11.69±1.16	11.10±0.37	10.21±0.11	12.29±0.60	11.63±1.08
NJAU_PW087	14.04±0.27	15.51±0.55	12.27±0.66	14.38±0.79	14.39±1.99	14.12±1.40

Line	100-seed weight (g) ± Standard deviation					
	2014LH	2015DT	2015JP	2018DT	2019DT	Mean
NJAU_PW088	14.71±0.20	15.21±0.81	14.18±1.19	13.29±1.18	13.19±0.16	14.12±1.08
NJAU_PW091	11.56±0.80	10.97±1.24	10.29±0.31	10.03±0.30	9.94±1.08	10.49±0.92
NJAU_PW092	11.83±0.84	10.26±0.37	10.60±0.49	8.60±0.00	10.15±1.47	10.55±1.15
NJAU_PW093	13.37±0.42	13.19±0.63	10.61±1.03	11.89±0.23	11.58±1.62	12.13±1.33
NJAU_PW094	12.99±0.15	11.09±1.27	11.00±0.35	10.75±0.37	10.57±2.65	11.32±1.49
NJAU_PW095	11.35±0.41	11.43±0.57	10.15±0.66	9.18±1.00	9.80±0.70	10.38±1.08
NJAU_PW096	13.25±0.45	10.72±0.31	9.68±0.60	10.04±0.84	.	10.92±1.54
NJAU_PW097	11.71±0.00	11.40±0.51	10.50±0.65	9.74±0.51	9.23±2.86	10.42±1.31
NJAU_PW098	14.12±0.68	11.01±0.74	10.91±0.65	9.49±0.75	10.24±0.94	11.15±1.75
NJAU_PW099	12.01±0.18	10.67±0.24	10.10±0.13	8.62±1.04	10.64±0.61	10.41±1.23
NJAU_PW100	13.42±0.03	12.50±0.81	10.91±1.75	10.94±0.83	12.54±0.70	12.05±1.23
NJAU_PW102	9.73±0.08	10.16±0.54	9.15±0.38	8.75±0.81	9.74±1.30	9.55±0.81
NJAU_PW103	14.62±0.98	14.06±0.08	12.96±1.18	11.66±1.17	14.01±1.44	13.46±1.42
NJAU_PW104	12.15±0.27	11.88±0.48	10.82±0.41	9.57±0.45	11.17±1.30	11.12±1.11
NJAU_PW105	13.71±0.88	14.27±0.99	9.02±0.76	14.23±0.75	13.30±0.84	12.91±2.16
NJAU_PW106	11.38±0.52	11.97±0.20	10.96±0.81	10.75±1.88	13.87±0.18	11.79±1.41
NJAU_PW107	15.46±0.39	14.03±0.10	12.21±0.96	12.36±1.55	13.07±1.44	13.51±1.53
NJAU_PW108	10.9±1.43	11.41±0.56	10.85±0.56	11.63±0.70	12.83±1.70	11.52±1.19
NJAU_PW109	12.06±0.62	11.41±0.38	9.97±0.34	9.10±0.29	10.65±1.41	10.64±1.24
NJAU_PW110	11.92±0.10	10.68±0.35	9.78±0.43	10.89±0.67	11.37±0.32	11.01±0.78
NJAU_PW111	14.07±0.24	13.04±0.43	12.58±0.15	13.47±1.40	9.57±0.90	12.55±1.75
NJAU_PW112	15.3±0.25	13.17±1.18	10.67±0.59	13.62±0.67	14.49±2.06	13.45±1.89
NJAU_PW113	10.78±0.94	11.81±0.00	9.64±0.82	9.05±0.81	11.82±0.92	10.44±1.35
NJAU_PW114	14.52±0.05	13.55±1.15	11.67±0.78	14.92±0.09	14.31±4.40	13.76±1.81
NJAU_PW115	12.01±1.10	12.10±0.83	11.97±0.19	10.52±0.47	11.36±1.03	11.59±0.92
NJAU_PW116	11.78±2.46	11.31±0.20	10.02±0.59	9.16±1.16	7.91±1.16	9.90±1.79
NJAU_PW117	12.11±0.20	10.92±0.87	9.71±0.94	9.75±0.56	6.71±0.64	9.84±1.95
NJAU_PW118	12.29±0.24	11.28±0.89	10.82±0.15	9.59±0.55	13.69±1.39	11.53±1.58
NJAU_PW119	11.31±0.49	11.04±0.27	11.05±0.30	9.41±0.40	9.06±0.05	10.33±1.03
NJAU_PW121	10.65±0.53	10.62±0.19	8.90±0.47	11.36±1.78	11.63±1.83	10.71±1.31
NJAU_PW122	12.51±0.66	12.65±1.15	11.20±0.91	11.85±2.67	9.44±2.15	11.53±1.87
NJAU_PW123	15.81±0.52	14.24±0.60	12.04±0.81	11.84±1.56	12.54±2.06	13.35±1.87
NJAU_PW124	12.45±1.32	12.87±1.38	12.37±0.6	11.87±0.71	14.24±1.02	12.76±1.22
NJAU_PW125	11.20±0.67	10.69±0.47	10.03±0.37	10.13±1.47	10.30±0.85	10.47±0.85
NJAU_PW126	14.42±0.05	12.24±0.59	11.86±0.60	11.54±0.73	12.97±1.21	12.61±1.23
NJAU_PW127	16.71±0.83	14.94±0.76	11.52±0.75	17.39±0.74	16.23±1.88	15.21±2.35
NJAU_PW128	9.11±1.06	10.92±0.28	8.88±0.14	9.67±0.53	8.90±0.62	9.54±0.97
NJAU_PW129	12.27±0.51	11.95±0.65	11.52±0.16	10.01±0.16	11.28±1.34	11.41±1.00
NJAU_PW130	13.86±0.65	12.64±0.06	12.55±0.25	12.64±1.41	9.01±0.00	12.63±1.50
NJAU_PW131	12.37±1.93	11.88±2.22	10.44±1.46	12.49±1.13	11.57±0.80	11.75±1.55
NJAU_PW132	14.98±0.30	14.01±0.70	13.08±1.20	11.51±0.45	13.95±3.26	13.43±1.87

	100-seed weight (g) ± Standard deviation					
Line	2014LH	2015DT	2015JP	2018DT	2019DT	Mean
NJAU_PW133	11.55±0.51	11.58±0.59	10.05±0.00	11.28±0.60	12.32±0.29	11.56±0.73
NJAU_PW134	12.34±0.65	14.24±0.31	12.82±0.31	10.99±0.16	13.35±0.53	12.75±1.18
NJAU_PW135	12.11±0.38	13.08±0.83	12.22±0.47	10.47±1.76	10.75±0.87	11.73±1.31
NJAU_PW136	13.23±1.16	11.52±0.91	9.84±0.14	10.62±1.43	11.32±0.22	11.31±1.41
NJAU_PW137	14.06±0.84	12.89±1.12	11.04±1.16	10.10±0.73	11.37±3.73	11.76±1.94
NJAU_PW138	15.50±1.29	15.19±1.84	12.48±1.03	12.39±1.14	13.38±1.50	13.79±1.80
NJAU_PW139	11.13±0.20	11.26±1.21	11.00±0.20	9.88±0.61	13.57±1.01	11.37±1.41
NJAU_PW140	14.45±0.47	12.29±0.19	11.46±0.62	11.81±0.84	12.16±0.10	12.44±1.17
NJAU_PW141	10.18±0.69	9.83±0.16	7.71±0.30	8.90±0.93	8.22±2.05	8.91±1.37
NJAU_PW142	14.46±0.13	13.91±0.67	10.94±1.95	11.21±0.86	15.57±0.78	13.22±2.10
NJAU_PW143	9.64±0.58	11.16±0.35	9.58±0.90	9.02±1.34	9.68±0.26	9.82±1.00
NJAU_PW144	14.09±1.09	13.50±0.51	12.48±0.71	12.56±1.52	14.03±2.14	13.33±1.33
NJAU_PW146	13.04±0.45	13.41±0.08	12.09±0.10	12.26±0.26	11.68±0.63	12.49±0.73
NJAU_PW148	11.85±0.28	11.36±0.42	11.47±0.18	9.91±0.10	11.16±1.38	11.24±0.85
NJAU_PW149	13.91±0.41	13.80±0.63	12.55±0.43	11.82±0.81	11.89±0.83	12.81±1.12
NJAU_PW150	12.17±0.25	12.09±0.43	11.25±0.56	10.87±0.33	11.75±0.93	11.62±0.67
NJAU_PW151	13.65±0.46	13.18±0.84	13.21±1.94	11.44±0.64	14.73±1.78	13.24±1.55
NJAU_PW152	14.09±0.33	11.87±1.36	9.55±0.48	9.36±0.42	12.50±0.15	11.47±1.95
NJAU_PW153	13.89±0.75	13.42±0.48	12.29±0.52	10.68±0.77	12.46±0.18	12.55±1.25
NJAU_PW154	13.90±0.00	11.64±0.81	10.86±0.41	9.82±1.05	8.83±2.11	10.57±1.79
NJAU_PW155	13.37±0.90	11.35±0.19	10.75±0.26	11.12±1.32	11.17±2.73	11.55±1.54
NJAU_PW156	18.08±1.52	19.95±1.32	17.86±0.43	13.00±1.88	19.09±0.57	17.59±2.72
NJAU_PW157	11.56±0.47	10.89±0.40	9.87±0.56	10.47±0.19	10.45±0.30	10.65±0.67
NJAU_PW158	13.16±0.88	12.55±0.56	11.27±0.49	11.77±0.27	11.69±3.67	12.12±1.33
NJAU_PW159	12.89±0.69	13.27±0.83	13.28±1.22	10.46±0.61	8.45±0.00	12.16±1.77
NJAU_PW160	11.77±0.91	12.14±1.17	11.15±0.63	10.88±0.14	11.69±0.00	11.50±0.83
NJAU_PW161	13.34±0.29	12.68±0.94	11.86±0.25	10.81±0.95	13.05±2.07	12.35±1.34
NJAU_PW163	14.44±0.42	12.42±0.66	12.58±0.74	10.95±1.78	13.19±2.31	12.71±1.66
NJAU_PW164	11.99±0.53	12.86±0.80	10.48±0.79	9.98±1.23	10.74±0.46	11.21±1.29
NJAU_PW165	14.28±0.75	14.21±0.26	13.97±1.24	11.92±0.44	12.89±1.51	13.49±1.22
NJAU_PW166	17.60±0.31	15.62±0.33	11.89±1.61	13.45±1.23	13.98±0.34	14.69±2.08
NJAU_PW167	14.05±0.41	14.37±0.30	13.63±0.56	13.74±0.98	13.15±0.67	13.79±0.68
NJAU_PW168	12.99±0.76	12.81±1.08	11.38±0.18	12.33±1.48	16.67±1.35	13.25±2.15
NJAU_PW169	14.05±0.69	15.66±0.41	13.74±0.30	13.30±1.94	12.72±1.30	13.91±1.44
NJAU_PW170	13.70±0.20	12.94±0.72	11.75±0.66	11.25±0.29	11.09±0.15	12.15±1.12
NJAU_PW171	11.89±0.70	13.39±0.84	12.32±1.00	10.88±0.14	10.77±2.73	12.01±1.40
NJAU_PW172	15.02±0.44	15.69±0.68	12.66±1.52	12.87±1.11	16.04±1.4	14.58±1.68
NJAU_PW174	14.33±0.84	15.12±0.45	13.22±0.01	10.43±0.75	12.57±2.14	12.97±1.97
NJAU_PW175	11.39±0.41	11.05±0.71	10.39±0.6	10.29±0.85	11.40±1.81	10.87±0.87
NJAU_PW176	17.70±0.21	16.95±0.94	14.56±0.00	12.46±1.00	14.88±0.11	15.42±2.13
NJAU_PW177	12.35±0.66	12.83±0.24	10.49±0.24	10.50±0.53	13.94±1.21	12.02±1.51

Line	100-seed weight (g) ± Standard deviation					
	2014LH	2015DT	2015JP	2018DT	2019DT	Mean
NJAU_PW178	11.00±0.34	11.16±0.19	11.07±0.30	10.23±1.05	10.60±1.43	10.81±0.78
NJAU_PW179	18.31±0.29	16.58±0.70	15.60±0.57	13.97±0.90	14.05±1.93	15.51±1.83
NJAU_PW180	11.24±0.48	11.14±0.31	10.93±0.07	9.14±0.21	10.07±1.15	10.47±0.99
NJAU_PW181	13.54±0.16	13.07±0.24	11.93±0.48	11.43±0.50	12.60±1.05	12.51±0.92
NJAU_PW182	15.03±0.48	13.18±0.56	13.07±0.67	12.89±0.87	11.33±0.00	13.37±1.19
NJAU_PW183	14.80±0.82	12.44±0.09	11.82±0.17	11.88±0.57	11.34±0.51	12.45±1.34
NJAU_PW184	14.23±0.27	14.24±0.21	12.59±1.21	13.53±0.57	14.72±1.35	13.83±1.09
NJAU_PW185	11.08±0.25	11.72±0.27	10.11±0.62	10.73±0.88	12.14±0.91	11.18±0.92
NJAU_PW186	13.66±0.87	12.15±0.37	11.88±0.29	10.55±0.75	11.28±1.92	11.95±1.32
NJAU_PW187	12.71±0.53	14.10±1.30	12.45±0.11	10.27±0.35	12.06±1.60	12.32±1.52
NJAU_PW188	14.66±0.16	12.76±0.15	11.67±0.68	12.78±1.11	14.87±0.87	13.35±1.40
NJAU_PW189	12.94±0.51	14.32±0.75	12.67±0.77	13.81±1.01	11.90±1.40	13.22±1.11
NJAU_PW190	14.26±0.26	13.53±0.59	11.29±1.61	12.48±1.34	11.17±1.24	12.64±1.53
NJAU_PW191	12.06±0.19	10.43±0.52	11.41±3.99	10.10±1.25	11.06±2.52	11.01±1.99
NJAU_PW192	14.01±0.24	12.98±0.19	13.19±0.81	12.55±0.54	13.96±0.73	13.29±0.73
NJAU_PW194	15.63±0.61	13.81±0.73	11.52±0.46	11.76±0.55	14.56±1.00	13.38±1.77
NJAU_PW195	14.27±0.83	12.95±0.36	9.97±0.33	10.88±0.21	13.64±2.72	12.34±2.02
NJAU_PW196	12.75±0.04	10.58±0.26	8.37±0.07	12.48±0.84	11.05±0.69	11.24±1.56
NJAU_PW197	13.15±1.79	12.91±0.55	11.47±0.13	12.59±0.41	11.73±0.25	12.38±0.88
NJAU_PW198	11.67±0.61	11.94±0.85	11.23±1.31	10.14±2.04	11.37±2.02	11.22±1.43
NJAU_PW199	13.68±0.70	13.77±0.42	11.54±0.97	10.53±0.23	12.52±1.02	12.4±1.46
NJAU_PW200	13.27±0.43	13.03±0.64	10.36±0.36	10.19±1.97	14.39±0.16	12.35±1.87
NJAU_PW201	13.86±0.17	14.37±0.62	13.69±0.25	10.71±0.34	10.61±1.53	12.65±1.82
NJAU_PW203	11.43±0.07	10.85±0.68	10.42±0.73	8.41±0.83	9.26±0.53	10.07±1.25
NJAU_PW204	13.41±0.83	12.77±0.25	9.35±0.46	12.04±1.40	13.22±0.98	11.98±1.73
NJAU_PW205	13.11±1.23	12.36±0.57	11.34±0.98	10.99±0.45	12.61±0.98	12.04±1.12
NJAU_PW206	11.52±0.07	10.48±0.38	8.08±0.44	7.81±0.82	8.65±1.76	9.31±1.68
NJAU_PW207	12.58±0.97	13.36±0.05	11.71±0.52	12.64±0.67	12.24±0.88	12.51±0.81
NJAU_PW208	10.65±0.57	11.27±0.37	9.62±0.93	12.79±1.99	12.91±1.50	11.45±1.67
NJAU_PW209	15.04±0.73	12.88±0.18	12.09±0.06	12.21±1.01	11.69±0.12	12.83±1.36
NJAU_PW210	13.08±0.67	11.86±0.12	10.69±0.50	12.17±0.30	13.89±1.31	12.34±1.28
NJAU_PW211	14.80±0.76	13.99±0.69	12.62±1.74	11.16±1.00	14.92±0.30	13.50±1.71
NJAU_PW212	11.95±0.04	11.57±0.36	10.81±0.11	10.35±0.78	9.28±0.00	11.04±0.93
NJAU_PW213	12.70±0.24	11.92±0.44	10.32±0.08	9.70±1.06	12.13±0.25	11.35±1.27
NJAU_PW214	15.45±1.17	13.22±0.46	11.19±1.29	10.92±0.65	10.55±2.46	12.27±2.24
NJAU_PW215	13.02±0.42	12.84±0.42	11.57±0.66	11.60±0.84	11.97±2.51	12.2±1.23
NJAU_PW216	11.55±1.01	11.58±0.65	10.54±0.74	10.21±0.55	10.89±1.22	10.91±0.90
NJAU_PW217	14.66±0.04	15.60±0.48	12.69±0.16	13.16±0.62	12.94±1.81	13.81±1.39
NJAU_PW219	13.05±1.65	14.90±0.59	13.91±2.26	11.96±0.00	10.00±0.82	12.72±2.22
NJAU_PW220	10.64±1.28	11.40±0.45	10.2±0.58	12.28±1.39	10.80±0.45	11.07±1.09
NJAU_PW221	14.32±0.65	14.77±0.71	13.80±0.51	12.03±0.57	.	13.73±1.21

Line	100-seed weight (g) ± Standard deviation					
	2014LH	2015DT	2015JP	2018DT	2019DT	Mean
NJAU_PW222	12.01±0.76	13.62±1.35	11.26±1.12	10.16±2.00	12.43±2.33	11.90±1.82
NJAU_PW224	11.26±0.74	10.19±0.86	9.27±0.64	9.23±0.62	12.36±1.20	10.33±1.34
NJAU_PW225	11.04±0.73	11.64±0.42	9.21±0.94	9.48±0.59	10.23±1.38	10.39±1.20
NJAU_PW226	14.13±0.67	14.66±2.17	14.15±2.22	12.43±0.42	10.36±0.43	13.15±2.05
NJAU_PW227	17.84±0.33	14.28±0.32	11.85±0.37	13.50±1.20	12.10±0.42	13.63±2.1
NJAU_PW228	13.72±0.67	15.06±1.10	13.32±1.23	10.62±1.27	13.35±2.17	13.27±1.76
NJAU_PW229	15.15±0.03	14.51±0.80	12.49±0.65	12.24±0.22	16.56±0.69	13.93±1.71
NJAU_PW230	15.57±0.20	18.17±1.43	13.19±0.56	13.75±1.61	16.97±1.47	15.70±2.17
NJAU_PW231	13.49±0.44	13.20±1.27	11.13±1.13	9.01±0.61	10.84±1.70	11.64±1.89
NJAU_PW232	11.14±0.95	10.87±0.56	10.52±0.74	10.00±1.13	8.97±1.83	10.24±1.24
NJAU_PW233	15.75±0.13	14.14±1.08	13.65±0.76	12.94±1.14	14.47±1.41	14.19±1.29
NJAU_PW234	12.74±1.30	13.34±0.53	12.21±1.67	14.50±0.72	13.50±1.04	13.32±1.18
NJAU_PW235	13.18±0.60	12.46±0.97	10.95±0.18	9.82±0.84	13.80±0.21	12.12±1.64
NJAU_PW236	13.78±1.11	13.02±0.52	10.73±0.35	11.83±0.01	10.32±0.05	11.95±1.50
NJAU_PW238	12.82±0.15	12.65±1.54	11.23±0.05	10.17±0.51	12.21±1.49	11.79±1.30
NJAU_PW239	14.78±0.58	13.90±0.62	12.59±0.72	12.47±1.06	12.85±0.48	13.28±1.12
NJAU_PW240	12.31±0.25	13.63±1.00	11.53±0.28	10.39±0.72	11.37±2.38	11.91±1.48
NJAU_PW241	11.91±0.45	11.75±0.30	10.56±0.12	9.67±0.17	9.27±1.64	10.63±1.28
NJAU_PW242	10.68±0.34	9.74±0.21	9.39±0.03	8.16±0.78	8.47±0.96	9.28±1.10
NJAU_PW244	13.61±0.48	12.74±0.36	10.38±1.50	10.50±0.64	12.74±0.24	12.00±1.51
NJAU_PW245	14.73±0.17	13.84±0.27	13.99±2.47	13.85±1.00	11.84±2.74	13.65±1.76
NJAU_PW246	12.53±0.87	11.2±1.19	11.07±0.20	9.31±0.36	13.23±0.00	11.20±1.44
NJAU_PW247	12.74±0.17	11.14±0.11	11.05±0.71	10.46±0.5	10.42±2.76	11.17±1.44
NJAU_PW248	.	12.26±0.13	15.32±0.00	10.91±0.6	15.34±2.53	13.62±3.18
NJAU_PW250	13.94±0.91	13.93±0.83	12.43±0.82	11.19±1.48	13.89±0.45	13.01±1.41
NJAU_PW251	15.22±0.43	15.52±0.72	14.18±1.36	13.73±0.29	13.19±1.38	14.37±1.22
NJAU_PW252	12.82±0.23	13.14±1.62	11.53±0.34	10.18±0.32	12.47±0.56	12.03±1.30
NJAU_PW253	14.35±0.56	14.44±0.81	12.51±0.26	11.28±0.48	12.04±2.36	12.93±1.64
NJAU_PW254	10.31±0.60	11.78±1.58	9.85±0.45	8.98±1.05	10.07±0.60	10.09±1.12
NJAU_PW255	15.75±0.44	14.27±0.44	12.98±0.38	13.13±0.86	13.43±1.22	13.91±1.23
NJAU_PW256	10.89±0.42	12.08±0.83	9.69±0.49	13.11±0.66	10.87±0.00	11.40±1.39
NJAU_PW257	11.20±0.17	12.25±1.09	10.13±0.64	10.51±0.22	12.06±1.08	11.23±1.07
NJAU_PW258	11.13±1.05	11.77±0.92	9.92±1.01	9.48±0.45	8.04±0.73	10.07±1.53
NJAU_PW259	10.26±0.70	9.28±0.61	9.67±0.92	10.03±0.81	11.09±0.24	10.07±0.87
NJAU_PW260	13.35±0.38	12.12±0.61	11.24±0.36	9.94±0.51	10.78±0.93	11.66±1.28
NJAU_PW261	15.21±0.80	15.74±0.87	12.63±0.43	13.93±0.78	14.78±1.30	14.38±1.37
NJAU_PW262	17.42±0.58	15.03±0.70	11.58±1.05	13.66±1.28	14.38±0.81	14.41±2.12
NJAU_PW263	8.77±0.15	11.28±2.05	10.17±1.51	9.81±2.38	10.63±1.21	10.13±1.64
NJAU_PW264	14.29±0.53	13.87±0.77	12.87±0.72	12.77±1.4	14.06±0.77	13.63±0.93
NJAU_PW265	13.23±1.94	12.28±1.13	12.29±2.25	10.57±0.31	13.13±1.30	12.24±1.64
NJAU_PW266	13.84±0.5	12.92±1.74	12.02±0.63	11.65±0.50	12.69±1.84	12.63±1.29

Line	100-seed weight (g) ± Standard deviation					
	2014LH	2015DT	2015JP	2018DT	2019DT	Mean
NJAU_PW268	18.20±0.32	15.56±0.65	13.70±0.59	13.25±2.25	14.96±1.93	15.13±2.16
NJAU_PW269	16.11±0.04	16.12±0.60	12.62±0.17	13.06±0.68	14.83±0.12	14.44±1.57
NJAU_PW270	14.21±0.29	12.32±0.52	11.58±0.24	10.79±0.86	13.64±1.79	12.51±1.53
NJAU_PW273	11.65±0.90	11.41±0.06	9.18±0.73	9.10±0.45	12.38±0.92	10.82±1.54
NJAU_PW274	10.81±1.63	10.89±0.45	10.95±0.52	11.71±0.49	11.15±3.08	11.1±1.17
NJAU_PW275	15.87±0.31	14.53±0.71	12.81±0.15	12.77±1.47	13.08±2.01	13.86±1.55
NJAU_PW276	12.96±1.10	13.61±1.68	12.42±2.11	12.38±0.49	12.89±0.58	12.85±1.23
NJAU_PW277	12.68±0.17	13.33±0.24	12.28±0.60	10.77±1.05	12.15±0.34	12.24±1.00
NJAU_PW279	14.35±0.62	12.80±0.57	10.96±1.29	11.87±0.34	13.61±1.34	12.72±1.47
NJAU_PW280	12.39±1.03	11.18±1.09	10.37±0.25	10.50±1.15	11.90±1.47	11.27±1.22
NJAU_PW281	12.35±0.69	12.82±0.89	11.03±0.66	11.12±0.69	10.81±1.61	11.57±1.18
NJAU_PW282	12.36±1.34	12.93±1.14	12.52±1.17	11.69±1.01	11.78±2.35	12.25±1.34
NJAU_PW283	12.63±0.55	12.43±0.50	11.90±0.37	10.85±0.50	15.51±0.40	12.66±1.66
NJAU_PW285	11.11±0.16	9.90±1.34	10.17±0.33	8.63±0.30	10.15±0.28	9.99±0.99
NJAU_PW286	18.49±0.04	17.55±0.98	15.32±0.59	14.47±1.67	15.62±1.79	16.29±1.85
NJAU_PW287	16.64±0.31	15.1±1.22	10.91±0.32	15.63±1.56	13.77±0.99	14.41±2.22
NJAU_PW288	13.25±0.42	13.02±0.31	11.06±0.60	11.72±0.57	12.42±1.82	12.29±1.08
NJAU_PW289	.	10.9±0.00	.	11.57±0.16	10.00±0.30	10.80±0.81
NJAU_PW291	12.05±0.33	12.57±0.16	12.23±0.18	11.84±1.11	11.72±2.89	12.05±1.26
NJAU_PW292	14.75±0.11	14.41±0.40	13.82±0.12	11.98±1.53	11.15±1.94	13.37±1.62
NJAU_PW293	13.99±0.93	13.60±1.22	12.50±0.25	12.35±0.49	11.72±3.85	12.86±1.51
NJAU_PW294	12.68±0.25	13.68±0.45	11.86±0.37	13.03±1.32	11.71±0.53	12.59±0.96
NJAU_PW295	11.12±0.16	11.97±0.18	10.19±0.72	9.30±0.48	11.97±3.00	11.03±1.59
NJAU_PW296	15.42±0.53	13.42±0.97	12.89±1.18	11.36±0.95	12.27±1.07	12.90±1.54
NJAU_PW297	12.70±0.00	11.66±0.42	10.43±1.42	8.83±0.23	11.00±0.56	10.84±1.31
NJAU_PW298	13.37±0.84	15.52±1.71	15.44±0.27	15.68±0.98	14.83±0.97	14.97±1.25
NJAU_PW299	14.27±0.21	11.81±0.96	11.89±0.35	13.11±1.26	13.78±1.06	13.05±1.24
NJAU_PW300	14.67±0.57	14.48±0.50	13.13±0.22	13.08±0.69	13.92±0.88	13.86±0.86
NJAU_PW301	12.59±0.57	12.57±0.22	10.28±0.59	9.93±1.35	12.54±0.35	11.58±1.40
NJAU_PW302	16.47±0.81	14.61±0.68	14.24±0.25	12.56±0.01	12.99±0.50	14.29±1.48
NJAU_PW303	15.06±0.27	15.55±0.89	13.97±1.41	12.31±0.46	7.12±0.32	12.84±3.35
NJAU_PW304	10.68±0.49	10.24±0.43	10.43±1.35	8.41±0.29	10.97±0.66	10.11±1.15
NJAU_PW305	15.54±0.37	16.34±1.53	12.91±1.07	13.50±0.62	13.59±1.69	14.38±1.69
NJAU_PW306	13.87±0.32	12.10±0.33	9.21±0.38	10.06±1.03	10.61±1.02	11.17±1.80
NJAU_PW307	15.91±0.76	15.31±0.41	14.44±0.72	12.77±0.22	12.44±1.64	14.27±1.61
NJAU_PW308	14.08±0.30	14.76±0.22	11.84±1.11	11.90±1.78	13.11±1.26	13.01±1.52
NJAU_PW309	11.39±0.20	12.13±0.11	11.19±0.49	10.77±0.65	10.29±1.37	11.15±0.88
NJAU_PW310	12.46±0.53	12.10±0.37	12.35±3.05	9.31±0.54	11.55±2.26	11.56±1.87
NJAU_PW311	12.25±0.61	12.57±0.33	11.18±1.29	10.48±0.56	11.45±0.41	11.54±0.99
NJAU_PW312	15.41±0.75	13.75±0.46	12.73±0.75	12.22±0.92	14.09±0.65	13.64±1.30
NJAU_PW313	13.97±0.52	12.77±1.02	10.91±0.82	10.40±0.71	12.58±0.38	12.13±1.48

Line	100-seed weight (g) ± Standard deviation					
	2014LH	2015DT	2015JP	2018DT	2019DT	Mean
NJAU_PW314	12.83±0.25	14.27±1.75	13.29±0.59	10.22±0.35	12.65±0.53	12.65±1.57
NJAU_PW315	14.73±1.09	14.28±1.07	14.03±1.47	13.34±0.61	12.27±2.85	13.73±1.63
NJAU_PW316	13.65±0.35	13.61±0.91	10.99±0.96	11.83±0.47	15.53±2.44	12.95±1.79
NJAU_PW317	19.32±2.72	18.89±1.08	16.32±1.67	12.77±0.62	15.62±0.89	16.45±2.79
NJAU_PW318	13.11±0.86	13.45±0.18	12.36±0.36	11.70±0.26	11.48±3.91	12.49±1.39
NJAU_PW319	10.00±0.36	9.81±0.24	9.56±0.64	9.45±0.96	12.07±0.58	10.18±1.13
NJAU_PW320	13.74±0.04	13.82±0.64	10.24±1.06	10.47±0.20	12.20±0.75	11.98±1.68
NJAU_PW321	14.40±0.53	14.64±2.87	12.48±0.43	14.31±0.74	15.52±2.76	14.27±1.86
NJAU_PW322	12.40±0.14	13.23±0.25	12.47±0.50	10.01±0.80	10.17±0.60	11.66±1.43
NJAU_PW323	10.85±0.00	11.32±0.47	11.24±0.73	10.61±2.61	11.28±0.57	11.09±1.19
NJAU_PW324	13.69±0.82	13.23±0.32	11.61±1.37	10.31±1.53	13.74±1.58	12.67±1.63
NJAU_PW325	12.30±1.17	15.69±1.70	11.36±0.11	13.42±1.68	14.40±1.74	13.43±1.98
NJAU_PW326	12.17±0.82	12.61±1.14	10.63±0.44	10.01±0.48	13.38±0.87	11.76±1.46
NJAU_PW327	14.11±0.48	12.58±0.46	10.75±0.68	11.54±1.78	13.05±0.37	12.41±1.44
NJAU_PW328	12.73±0.26	12.07±1.00	9.59±0.44	9.90±0.72	10.85±1.47	11.03±1.46

The 100-seed weight in 2014LH, 2015DT, 2015JP, 2018DT, 2019DT and the average value across five environments, respectively. 2014LH, experiment at Liuhe in 2014; 2015DT, experiment at Dangtu in 2015; 2015JP, experiment at Jiangpu in 2015; 2018DT, experiment at Dangtu in 2018; 2019DT, experiment at Dangtu in 2019. Data represents the mean ± standard deviation (SD) of three replications under each environment. Mean, the mean ± SD of 100-seed weight across five environments of 2014LH, 2015DT, 2015JP, 2018DT and 2019DT. “.”, the data was missing for the line.

Supplementary Table 3 Summary of the sequencing data for two parents.

Sample	Raw data(bp)	Clean data(bp)	Effective rate (%)	Error rate (%)	Q20 (%)	Q30 (%)	GC content (%)
PI	12,648,198,300	12,621,300,900	99.79	0.03	97.22	92.36	36.08
WH	11,022,993,600	11,004,099,900	99.83	0.03	97.82	93.64	35.91

Sample, the sample name. Raw data(bp), the total number of bases in the raw data, the unit is bp. Clean data, the total number of bases in the clean data, the unit is bp. Effective rate (%), the ratio of the clean data over raw data. Error rate (%), base error rate. Q20/Q30, the percentage of bases with a clean data quality value greater than or equal to Q20 or Q30. GC content, the GC content of the clean data, that is, the percentage of the G and C bases in the clean data to the total bases.

Supplementary Table 4 Summary of mapping rates for two parents.

Sample	Clean reads	Mapped reads	Mapped rate	Coverage 1×	Coverage 4×
PI	84142006	83225679	98.91%	98.12%	90.86%
WH	73360666	72599526	98.96%	96.98%	86.36%

Sample, the sample name. Clean reads, the total number of pair-end reads in the clean data. Mapped rate, the ratio of the mapped reads over clean reads. Coverage 1×, the ratio of the at least one base in the reference genome. Coverage 4×, the ratio of the at least four bases in the reference genome.

Supplementary Table 5 Statistics of the bin marker information for the genetic linkage map in the NJPW-RIL population.

Chr.	No. of bins	Genetic distance of bin markers		Physical length of bin markers	
		Total (cM)	Mean (cM)	Total (Mb)	Mean (Mb)
1	236	145.85	0.62	56.83	0.24
2	273	210.79	0.77	48.58	0.18
3	256	183.10	0.72	45.78	0.18
4	215	176.02	0.82	52.39	0.24
5	204	163.06	0.80	42.23	0.21
6	268	249.47	0.93	51.42	0.19
7	224	158.07	0.71	44.63	0.20
8	274	182.85	0.67	47.84	0.17
9	251	183.94	0.73	50.19	0.20
10	232	170.25	0.73	51.57	0.22
11	205	186.16	0.91	34.77	0.17
12	184	128.85	0.70	40.09	0.22
13	302	209.82	0.69	45.87	0.15
14	227	149.00	0.66	49.04	0.22
15	245	169.03	0.69	51.76	0.21
16	200	155.16	0.78	37.89	0.19
17	213	152.39	0.72	41.64	0.20
18	262	167.45	0.64	58.02	0.22
19	246	191.98	0.78	50.75	0.21
20	185	143.46	0.78	46.94	0.25
**Together**	**4702**	**3476.68**	**0.74**	**948.22**	**0.20**

Chr., chromosome. No. of bins, number of bin markers. Total (cM), total genetic distance of each chromosome. Mean (cM), average genetic distance between two adjacent markers. Total (Mb), total physical distance of each chromosome. Mean (Mb), average physical distance between two adjacent markers.

Supplementary Table 6 The quantitative trait loci (QTL) for 100-seed weight identified in the NJPW-RIL population under multiple environments.

QTL	Env.	Chr.	Position (cM)	1-LOD interval (cM)	Flanking markers	Physical region (bp)	LOD	R^2 (%)	A	Reported QTL/genes
qSw-2-1	2015JP	2	207.71	203.70-208.60	bin505-bin511	46512310-47442266	3.68	3.50	0.32	*Seed weight 37-10, 49-9,50-12*
	2014LH	2	208.61	206.40-210.10	bin507-bin513	46848846-47680291	4.28	3.60	0.37	
qSw-4-1	2014LH	4	46.31	45.10-47.30	bin840-bin843	7839751-8194889	9.20	8.59	-0.57	*Seed weight 7-3, 54-1, 54-2*
qSw-4-2	2014LH	4	54.11	52.90-54.90	bin849-bin852	8845864-9221467	9.06	8.46	-0.57	*Seed weight 47-3, 50-9*
	MEAN	4	55.31	54.60-56.40	bin851-bin854	9043869-9444748	5.72	4.91	-0.35	
qSw-4-3	2014LH	4	64.11	61.70-64.30	bin860-bin871	9940392-12583674	4.50	4.35	-0.41	*Seed weight 5-2, 20-2, 47-3, 50-9*
qSw-5-1	2015JP	5	151.41	149.90-152.90	bin1187-bin1193	40029120-40801549	3.65	3.48	0.31	*Seed weight 7-3, 10-1, 34-9*
qSw-5-2	2015JP	5	159.51	158.10-160.70	bin1198-bin1203	41233646-41849262	4.87	4.62	0.36	*Seed weight 10-1*
qSw-7-1	MEAN	7	1.21	0.40-2.00	bin1482-bin1486	1-646519	4.04	3.21	0.28	This study
qSw-7-2	2014LH	7	24.31	23.40-25.10	bin1516-bin1521	3857316-4545812	6.13	5.58	0.47	*Seed weight 49-15*
qSw-7-3	MEAN	7	73.01	72.10-74.80	bin1571-bin1577	9922749-10726768	4.38	3.77	0.31	*Seed weight 49-15*
qSw-7-4	MEAN	7	82.11	80.40-84.30	bin1593-bin1599	14780661-15502117	5.29	4.52	0.33	*Seed weight 45-5*
qSw-7-5	2014LH	7	87.51	86.00-88.20	bin1600-bin1604	15502118-16009213	3.69	3.13	0.35	*Seed weight 45-5*
	2015DT	7	89.21	88.60-89.50	bin1603-bin1604	15833869-16009213	7.97	9.21	0.87	
qSw-7-6	2019DT	7	91.51	90.10-92.00	bin1604-bin1608	15886272-16442953	4.28	5.13	0.45	*Seed weight 45-5*
	MEAN	7	91.51	90.60-92.00	bin1605-bin1608	16009214-16442953	5.06	4.34	0.33	
qSw-7-7	2015DT	7	96.41	95.40-97.10	bin1610-bin1613	16598711-17109097	4.07	4.90	0.41	*Seed weight 45-5, 12-4*
qSw-7-8	2015DT	7	101.41	101.40-101.80	bin1619-bin1621	17699311-18067493	5.67	5.96	-0.69	*Seed weight 45-5, 12-4*
qSw-8-1	2015JP	8	75.81	72.10-77.70	bin1817-bin1820	14648877-15199558	5.96	6.56	0.43	*Seed weight 34-13, 35-1, 49-1*
qSw-8-2	2015JP	8	82.81	82.00-84.30	bin1827-bin1833	15818764-16521471	3.92	4.08	0.34	*Seed weight 34-13, 35-1, 49-1*

QTL	Env.	Chr.	Position (cM)	1-LOD interval (cM)	Flanking markers	Physical region (bp)	LOD	R^2 (%)	A	Reported QTL/genes
qSw-8-3	2014LH	8	123.51	122.90-124.00	bin1892-bin1896	35137650-35711298	3.87	3.27	0.36	*Seed weight 10-2, 34-1, 34-13, 36-1, 37-8, 49-1*
qSw-8-4	2014LH	8	129.81	129.60-131.80	bin1907-bin1913	37210320-39009553	4.05	3.42	0.37	*Seed weight 10-2, 34-1, 34-13, 36-1, 37-8, 49-1*
qSw-8-5	2014LH	8	147.71	147.50-148.60	bin1943-bin1947	42815986-43303496	4.97	4.17	0.41	*Seed weight 34-1, 34-13, 36-1, 49-1*
qSw-10-1	2014LH	10	160.91	159.80-164.60	bin2469-bin2474	49631239-50229424	3.67	3.11	0.35	This study
qSw-11-1	2015DT	11	83.31	75.80-90.30	bin2571-bin2625	11028820-26044407	3.73	3.99	0.37	*Seed weight 10-3, 11-1, 20-1, 20-3, 20-4, 32-1, 36-11*
qSw-12-1	2019DT	12	95.21	93.00-98.00	bin2826-bin2835	34286458-35448712	4.64	5.30	-0.46	*Seed weight 13-8, 34-4, 35-4 , 36-4, 41-1, 50-15*
qSw-14-1	2015JP	14	119.61	116.60-121.90	bin3374-bin3381	45010021-45825175	3.58	3.40	-0.31	This study
qSw-16-1	2015JP	16	110.11	109.10-112.70	bin3815-bin3817	30742438-31338518	4.73	4.80	0.37	This study
	MEAN	16	111.11	109.40-114.60	bin3815-bin3817	30742438-31338518	3.74	3.22	0.29	
qSw-17-1	2018DT	17	0.01	0.00-1.40	bin3874-bin3877	1-2249904	6.84	7.34	0.44	*Seed weight 3-1*
	MEAN	17	1.91	1.40-4.80	bin3876-bin3880	1948442-2537479	3.97	3.16	0.28	
qSw-17-2	2019DT	17	125.91	125.20-127.70	bin4060-bin4063	38428942-38849244	4.86	5.58	0.47	*Seed weight 47-2, 49-10, 50-3*
qSw-17-3	2014LH	17	138.91	136.60-140.80	bin4070-bin4075	39546586-40125043	3.81	3.41	0.36	*Seed weight 47-2, 50-3*
	MEAN	17	138.91	136.40-140.90	bin4070-bin4075	39546586-40125043	3.78	3.01	0.27	
qSw-19-1	**2015JP**	**19**	**135.11**	**134.20-136.90**	**bin4518-bin4523**	**40142359-40727409**	**10.89**	**11.60**	**0.57**	***Seed weight 35-7***
qSw-19-2	2015DT	19	149.11	148.90-149.50	bin4536-bin4539	42041403-42389415	8.13	8.38	0.54	*Seed weight 5-1, 17-1 ,34-7, 35-7*
	MEAN	19	149.11	148.40-149.30	bin4536-bin4539	42041403-42389415	7.80	7.55	0.43	
	2018DT	19	150.31	148.30-150.60	bin4536-bin4540	42041403-42537473	4.37	4.93	0.36	
qSw-19-3	2014LH	19	153.01	152.50-153.70	bin4544-bin4545	42928838-43135876	9.04	8.77	0.58	*Seed weight 5-1, 15-7, 17-1, 34-7, 35-7, 36-7, 43-4*
qSw-19-4	2018DT	19	155.91	155.10-156.30	bin4547-bin4550	43236796-43622656	8.51	9.27	0.49	*Seed weight 5-1, 7-7, 15-7, 17-1, 34-7, 35-7, 36-7, 43-4*
qSw-19-5	**2015DT**	**19**	**158.61**	**157.80-159.50**	**bin4552-bin4555**	**43732884-44300740**	**11.36**	**11.42**	**0.62**	***Seed weight 7-7, 17-1, 43-4***

QTL	Env.	Chr.	Position (cM)	1-LOD interval (cM)	Flanking markers	Physical region (bp)	LOD	R^2 (%)	A	Reported QTL/genes
qSw-19-5	**MEAN**	**19**	**158.61**	**157.50-159.60**	**bin4551-bin4555**	**43622657-44300740**	**14.59**	**13.43**	**0.58**	
	**2014LH**	**19**	**159.01**	**157.90-160.60**	**bin4552-bin4556**	**43732884-44410494**	**11.39**	**10.84**	**0.64**	
	**2019DT**	**19**	**159.01**	**158.00-160.60**	**bin4552-bin4556**	**43732884-44410494**	**8.08**	**9.52**	**0.62**	
qSw-19-6	2018DT	19	164.11	163.40-165.00	bin4562-bin4566	44958395-45485310	6.07	6.73	0.42	*Seed weight 7-7, 43-4*
qSw-19-7	2019DT	19	168.51	167.80-168.90	bin4566-bin4569	45414083-45851697	5.44	6.53	0.51	*Seed weight 7-7*
qSw-20-1	2018DT	20	31.51	29.90-32.40	bin4634-bin4638	2836033-3802360	6.28	6.75	0.42	*Seed weight 8-1, 34-5, 35-5*
	2015JP	20	33.61	33.40-33.90	bin4638-bin4641	3205982-4399985	3.61	4.23	0.35	
	MEAN	20	33.61	32.90-34.10	bin4638-bin4639	3205982-4227408	5.54	6.23	0.39	
qSw-20-2	**2014LH**	**20**	**37.61**	**37.00-37.80**	**bin4647-bin4649**	**5597957-11292631**	**13.48**	**13.33**	**0.72**	***Seed weight 8-1, 34-5, 35-5***
	**2015JP**	**20**	**38.71**	**38.30-40.20**	**bin4651-bin4663**	**11587440-24512220**	**6.63**	**7.58**	**0.47**	
	**2018DT**	**20**	**38.71**	**37.90-39.20**	**bin4649-bin4659**	**8901965-23163073**	**6.55**	**7.02**	**0.43**	
	**MEAN**	**20**	**38.71**	**38.20-39.50**	**bin4651-bin4660**	**11587440-23436726**	**8.82**	**9.70**	**0.50**	
	**2015DT**	**20**	**39.01**	**38.80-39.70**	**bin4654-bin4661**	**12361923-23597535**	**4.30**	**4.15**	**0.38**	
qSw-20-3	**2015DT**	**20**	**43.91**	**43.50-44.20**	**bin4673-bin4678**	**28796204-33344706**	**5.31**	**5.08**	**0.43**	***Seed weight 9-1***
	**2015JP**	**20**	**43.91**	**43.50-45.00**	**bin4673-bin4680**	**28796204-33545198**	**7.69**	**8.70**	**0.50**	
	**MEAN**	**20**	**43.91**	**43.50-44.00**	**bin4673-bin4676**	**28796204-32423296**	**9.78**	**10.66**	**0.52**	
	**2018DT**	**20**	**44.91**	**44.2-47.3**	**bin4676-bin4681**	**32057618-33608788**	**6.69**	**7.17**	**0.44**	
	**2014LH**	**20**	**45.91**	**44.20-46.90**	**bin4676-bin4681**	**32057618-33608788**	**14.92**	**15.03**	**0.76**	
qSw-20-4	MEAN	20	52.51	51.30-53.50	bin4686-bin4689	34022294-34449444	5.39	6.07	0.39	*Seed weight 9-1, 24-3,29-5, 34-5, 35-5, 36-5, 50-13*

QTL, quantitative trait loci identified for 100-seed weight in the NJPW-RIL population; *qSw-2-1*, *q* represents the abbreviation of QTL, *Sw* represents the 100-seed weigh, *-2* represents Chromosome 2, and *-1* represents its order on the chromosome according to its physical position. QTL detected in different environments at the same or overlapping marker intervals were considered as the same QTL. Env., environment. 2014LH, 2015DT, 2015JP, 2018DT, 2019DT and MEAN represent 2014Liuhe, 2015Dangtu, 2015Jiangpu, 2018Dangtu, 2019Dangtu and the average

values of 100-seed weight across five environments, respectively. Chr., Chromosome. Position, position of the QTL. 1-LOD interval, 1-LOD support confidence intervals (confidence interval length). Physical region, the physical position on Wm82 v2.1 genome in bp. R^2, the percentage of phenotypic variation explained by each QTL. LOD, the logarithm of odds (LOD) value at the peak likelihood of the QTL; the LOD thresholds were calculated from a 1000-permutation test (at the significance level of 0.05) by using CIM model in WinQTLCart2.5 Software, which were 3.60, 3.50, 3.50, 3.50, 3.70 and 3.60 for 2014LH, 2015DT, 2015JP, 2018DT, 2019DT and MEAN, respectively. A, additive effect, the estimated additive effect of the alleles from the maternal parent PI. Reported QTL/genes, the physical location overlaps with the previously reported QTL in Soybase (www.soybase.org). The major QTL were written in bold font, which were identified in at least three environments or $R^2 \geq 10\%$, for 100-seed weight in this study.

Supplementary Table 7 Thirty-four possible candidate genes and annotations related to 100-seed weight.

QTL	Gene ID	Functional annotation
qSw-19-1	*Glyma.19G142700*	GO:0016020, membrane; GO:0016021, integral component of membrane.
	Glyma.19G143200	GO:0003677, DNA binding; GO:0008270, zinc ion binding.
	Glyma.19G143300	GO:0004672, protein kinase activity; GO:0004674, protein serine/threonine kinase activity; GO:0005515, protein binding; GO:0005524, ATP binding; GO:0006468, protein phosphorylation; GO:0016772, transferase activity, transferring phosphorus-containing groups.
	Glyma.19G145000	GO:0003723, RNA binding; GO:0003735, structural constituent of ribosome; GO:0005622, intracellular; GO:0005840, ribosome; GO:0006412, translation; GO:0015935, small ribosomal subunit.
	Glyma.19G145100	GO:0003723, RNA binding; GO:0003735, structural constituent of ribosome; GO:0005622, intracellular; GO:0005840, ribosome; GO:0006412, translation; GO:0015935, small ribosomal subunit.
qSw-19-5	*Glyma.19G176700*	Unannotated
	Glyma.19G178800	GO:0003735, structural constituent of ribosome; GO:0005622, intracellular; GO:0005840, ribosome; GO:0006412, translation; GO:0015935, small ribosomal subunit.
	Glyma.19G179700	GO:0003677, DNA binding; GO:0005634, nucleus; GO:0006355, regulation of transcription, DNA-templated.
	Glyma.19G180700	Unannotated
	Glyma.19G181000	GO:0008152, metabolic process; GO:0008168, methyltransferase activity.
	Glyma.19G181300	GO:0005215, transporter activity; GO:0006810, transport; GO:0006833, water transport; GO:0016020, membrane; GO:0016021, integral component of membrane; GO:0055085, transmembrane transport.
	Glyma.19G182000	GO:0003824, catalytic activity; GO:0008836, diaminopimelate decarboxylase activity; GO:0009089, lysine biosynthetic process via diaminopimelate; GO:0003824, catalytic activity; GO:0008836, diaminopimelate decarboxylase activity; GO:0009089, lysine biosynthetic process via diaminopimelate.
	Glyma.19G182400	GO:0004001, adenosine kinase activity; GO:0006166, purine ribonucleoside salvage.
	Glyma.19G182800	GO:0043565, sequence-specific DNA binding
qSw-20-2	*Glyma.20G045500*	GO:0004869, cysteine-type endopeptidase inhibitor activity
	Glyma.20G048700	GO:0004252, serine-type endopeptidase activity; GO:0006508, proteolysis.
	Glyma.20G048900	GO:0005509, calcium ion binding
	Glyma.20G052500	GO:0003676, nucleic acid binding; GO:0005515, protein binding.

QTL	Gene ID	Functional annotation
qSw-20-2	***Glyma.20G053200***	GO:0005488, binding; GO:0006520; cellular amino acid metabolic process; GO:0055114, oxidation-reduction process.
	Glyma.20G054300	GO:0044780, bacterial-type flagellum assembly
	Glyma.20G054400	GO:0005488, binding
	Glyma.20G055900	GO:0003871, 5-methyltetrahydropteroyltriglutamate-homocysteine S-methyltransferase activity; GO:0008270, zinc ion binding; GO:0008652, cellular amino acid biosynthetic process; GO:0009086, methionine biosynthetic process.
	Glyma.20G058100	GO:0005515, protein binding.
	Glyma.20G062700	GO:0006869, lipid transport.
	Glyma.20G065300	GO:0016020, membrane.
qSw-20-3	*Glyma.20G078000*	Unannotated
	Glyma.20G081600	GO:0009733, response to auxin; GO:0040029, regulation of gene expression, epigenetic.
	Glyma.20G083300	GO:0003723, RNA binding; GO:0003735, structural constituent of ribosome; GO:0005622, intracellular; GO:0005840, ribosome; GO:0006412, translation; GO:0015935, small ribosomal subunit.
	Glyma.20G084000	GO:0000398, mRNA splicing, via spliceosome; GO:0005634, nucleus; GO:0005732, small nucleolar ribonucleoprotein complex; GO:0006396, RNA processing.
	Glyma.20G088200	GO:0016020, membrane; GO:0016021, integral component of membrane.
	Glyma.20G089400	GO:0005488, binding; GO:0005515, protein binding.
	Glyma.20G090700	GO:0003677, DNA binding; GO:0005515, protein binding; GO:0006355, regulation of transcription, DNA-templated.
	Glyma.20G091300	GO:0016021, integral component of membrane.
	Glyma.20G092200	GO:0016020, membrane; GO:0016021, integral component of membrane.

QTL, quantitative trait loci for 100-seed weight identified in the NJPW-RIL population. The gene names in bold font represent the potential candidate genes which were selected based on the known signaling pathways that control seed size, including ubiquitin-proteasome pathway, G-protein signaling, mitogen-activated protein kinase (MAPK) signaling, phytohormones as well as transcriptional regulators (Li et al. 2019).

Supplementary Table 8 Sequence variation of six candidate genes in two parental lines based on the re-sequencing data.

Gene ID	Chr.	Start	End	SNPs/Indel	SNP/Indel position	PI	WH	Location
Glyma.19G143300	19	40439199	40442902	SNP1	40437238	A	T	upstream
				SNP2	40437303	C	T	upstream
				SNP3	40437648	T	C	upstream
				Indel1	40438022	-	T	upstream
				SNP4	40439221	T	G	UTR5
				SNP5	40439270	C	T	UTR5
				SNP6	40439334	T	A	UTR5
				SNP7	40439503	T	C	exonic
				SNP8	40439623	A	G	exonic
				SNP9	40441635	C	T	exonic
				SNP10	40442807	C	T	UTR3
Glyma.19g182400	19	44132739	44137801	SNP1	44133984	G	C	intronic
				SNP2	44133986	G	T	intronic
Glyma.20g081600	20	30778586	30779164	SNP1	30776648	A	G	upstream
				SNP2	30776844	A	C	upstream
				SNP3	30777472	G	A	upstream
				SNP4	30777921	A	C	upstream
				SNP5	30777993	A	G	upstream

Chr., Chromosome. The sequence variations were detected using the whole genome sequencing at 30× depth of the two parents (PI and WH) in another study (unpublished) in our lab.

Supplementary Table 9 The 100-seed weight of soybean accessions carrying two different CDS types of *Glyma.19G143300*.

CDS type	Sample ID	Accessions	HSW (g)
CDS1	IGDB-TZX-0061	Ji Dou No.16	23.30
CDS1	IGDB-TZX-012	Lincoln	15.50
CDS1	IGDB-TZX-0121	PuDongDaHuangDou	23.10
CDS1	IGDB-TZX-0321	Amsoy	16.70
CDS1	IGDB-TZX-0401	GuanYunMaoYeDou	7.30
CDS1	IGDB-TZX-047	Pi Xian Hong Mao You	13.20
CDS1	IGDB-TZX-0521	QingPiPingDingXiang	15.50
CDS1	IGDB-TZX-0601	NanNong493-1	19.50
CDS1	IGDB-TZX-0621	Hao Cai No.1	18.00
CDS1	IGDB-TZX-0641	Wu Xing No.3	23.30
CDS1	IGDB-TZX-0651	Jiu Nong No.27	20.00
CDS1	IGDB-TZX-0701	XuDou13	21.50
CDS1	IGDB-TZX-071	Conrad	16.70
CDS1	IGDB-TZX-081	Zane	17.40
CDS1	IGDB-TZX-0821	ZhongHuang6	20.20
CDS1	IGDB-TZX-090	Ping ding xiang	17.70
CDS1	IGDB-TZX-0921	Shan Ning No.7	16.50
CDS1	IGDB-TZX-0981	YuDou8Hao	23.00
CDS1	IGDB-TZX-100	L62-1251	16.70
CDS1	IGDB-TZX-1011	Zong Mao Bian Jing	12.80
CDS1	IGDB-TZX-1061	Ji Dou No.17 *	18.00
CDS1	IGDB-TZX-112	Higan	23.50
CDS1	IGDB-TZX-117	TuErYan	20.50
CDS1	IGDB-TZX-119	KeFeng15Hao	25.00
CDS1	IGDB-TZX-1251	He Feng No.30	18.20
CDS1	IGDB-TZX-128	Ke Dou No.1	19.10
CDS1	IGDB-TZX-136	ZhongHuang34	21.50
CDS1	IGDB-TZX-1421	TaiNong15Hao	13.50
CDS1	IGDB-TZX-150	Tokei 423	21.30
CDS1	IGDB-TZX-1751	Sui Nong No.22	22.00
CDS1	IGDB-TZX-189	ZhouDou18	18.90
CDS1	IGDB-TZX-201	KAS 230-4	26.50
CDS1	IGDB-TZX-2061	JiDou18	23.90
CDS1	IGDB-TZX-229	ZhenYuanBaYueZha	18.20
CDS1	IGDB-TZX-250	NS-16	14.30
CDS1	IGDB-TZX-2521	Hua Dou No.2	26.00
CDS1	IGDB-TZX-254	ChangNong13	20.00
CDS1	IGDB-TZX-2601	Su Dou No.1	18.50
CDS1	IGDB-TZX-263	Corsoy	15.40
CDS1	IGDB-TZX-269	Ji Yuan Shui Bai Dou	11.00
CDS1	IGDB-TZX-2701	XuDou9Hao	23.50
CDS1	IGDB-TZX-2721	Gao Feng No.1	17.00
CDS1	IGDB-TZX-278	ZhouDou11	22.50
CDS1	IGDB-TZX-2811	Jin Dou No.39 -1	42.30
CDS1	IGDB-TZX-2821	Zhong Huang No.30	18.10
CDS1	IGDB-TZX-285	JinDa78Hao	21.00

CDS type	Sample ID	Accessions	HSW (g)
CDS1	IGDB-TZX-288	Wo Yang Da Jian Ke	19.00
CDS1	IGDB-TZX-296	Fang Zheng Wa Shi Dou	8.00
CDS1	IGDB-TZX-3021	ZhuYeQing	8.80
CDS1	IGDB-TZX-311	Mejiro	16.40
CDS1	IGDB-TZX-317	Di Liu Huang Dou -2	10.70
CDS1	IGDB-TZX-327	Si Li Yuan	20.00
CDS1	IGDB-TZX-3421	Кубанская	11.90
CDS1	IGDB-TZX-3571	Zao Shu No.17	18.00
CDS1	IGDB-TZX-359	JiHuang103	19.20
CDS1	IGDB-TZX-369	JIU YUE QING	12.20
CDS1	IGDB-TZX-370	ZDD05996	17.50
CDS1	IGDB-TZX-3701	BAI PI ZI	22.80
CDS1	IGDB-TZX-3721	HeDou12	24.80
CDS1	IGDB-TZX-391	Maple Glen	19.70
CDS1	IGDB-TZX-3961	Han6192	18.60
CDS1	IGDB-TZX-398	JieJieSi	34.50
CDS1	IGDB-TZX-402	Mukden	16.10
CDS1	IGDB-TZX-405	HeDou16	16.70
CDS1	IGDB-TZX-4222	Enrei	27.30
CDS1	IGDB-TZX-4241	HeiNong64	21.00
CDS1	IGDB-TZX-4301	SuiXiaoLiDou1Hao	10.00
CDS1	IGDB-TZX-433	T122	15.60
CDS1	IGDB-TZX-445	Zhong Huang No.13 -1	24.10
CDS1	IGDB-TZX-4471	Yu Dou No.22 *	19.30
CDS1	IGDB-TZX-469	Kai Feng Qing Dou	11.60
CDS1	IGDB-TZX-471	Preston	15.60
CDS1	IGDB-TZX-4731	KenNong4Hao	20.00
CDS1	IGDB-TZX-480	Williams	19.00
CDS1	IGDB-TZX-4801	HeiQiHuangDou	22.20
CDS1	IGDB-TZX-481	Clark	16.90
CDS1	IGDB-TZX-489	Zheng No.59	16.80
CDS1	IGDB-TZX-4951	MengDou11Hao	19.50
CDS1	IGDB-TZX-504	S-56	18.20
CDS1	IGDB-TZX-5111	ZaoZhuangLengDaDou	22.10
CDS1	IGDB-TZX-5221	Tai Wan Yi Hao	15.10
CDS1	IGDB-TZX-537	Xi Chuan Ji Wo Huang	9.10
CDS1	IGDB-TZX-539	Tie Jia Hei	12.50
CDS1	IGDB-TZX-5401	SuZhouWuYueMao	17.10
CDS1	IGDB-TZX-544	Ji Yu No.90	21.00
CDS1	IGDB-TZX-5731	Ken Nong No.5	18.90
CDS1	IGDB-TZX-5751	NenFeng16	25.60
CDS1	IGDB-TZX-579	He Nan Zao Feng No.1	12.00
CDS1	IGDB-TZX-5801	Da Li Hei	17.60
CDS1	IGDB-TZX-581	Perry	17.90
CDS1	IGDB-TZX-591	Young	12.50
CDS1	IGDB-TZX-605	ShengDou9Hao	25.00
CDS1	IGDB-TZX-6071	JingHuang3Hao	20.00
CDS1	IGDB-TZX-620	L62-667	15.90

CDS type	Sample ID	Accessions	HSW (g)
CDS1	IGDB-TZX-621	Shirome	11.80
CDS1	IGDB-TZX-631	Wu chang hei dong dou	21.90
CDS1	IGDB-TZX-6421	WDD01252	14.50
CDS1	IGDB-TZX-649	JiDou7Hao	18.50
CDS1	IGDB-TZX-657	NiDou	10.50
CDS1	IGDB-TZX-679	Yu Dou No.10	25.00
CDS1	IGDB-TZX-6821	ZhongHuang43	17.50
CDS1	IGDB-TZX-6881	Zhong Huang No.66	19.00
CDS1	IGDB-TZX-6901	Ji Ti No.2	19.80
CDS1	IGDB-TZX-691	Lloyd	8.70
CDS1	IGDB-TZX-6941	HeiNong2Hao	21.00
CDS1	IGDB-TZX-7021	ShanDou125	22.50
CDS1	IGDB-TZX-7222	KenNong14	20.00
CDS1	IGDB-TZX-726	Zhong Huang No.28	22.00
CDS1	IGDB-TZX-731	Xiao li huang	12.20
CDS1	IGDB-TZX-735	Cang Dou No.11	19.20
CDS1	IGDB-TZX-757	Jing Huang 35 Yi	12.50
CDS1	IGDB-TZX-7601	XuDou10Hao	23.00
CDS1	IGDB-TZX-7631	SuiNong30	17.00
CDS1	IGDB-TZX-764	Liao Dou No.22	21.40
CDS1	IGDB-TZX-768	Fen Dou No.56	23.40
CDS1	IGDB-TZX-7711	Jin Da No.74	20.40
CDS1	IGDB-TZX-7761	CangDou10Hao	23.00
CDS1	IGDB-TZX-784	Sui Nong No.10	20.00
CDS1	IGDB-TZX-7901	LiaoDou10Hao	23.00
CDS1	IGDB-TZX-7921	Jin Dou No.23 -1*	23.75
CDS1	IGDB-TZX-8001	SuiWuXing1Hao	18.70
CDS1	IGDB-TZX-8041	DongNong59	19.00
CDS1	IGDB-TZX-8211	Qi Si Mi	21.10
CDS1	IGDB-TZX-8222	Ken Feng No.19	18.50
CDS1	IGDB-TZX-8321	Lincoln	15.50
CDS1	IGDB-TZX-8371	YuDou12	20.00
CDS1	IGDB-TZX-8401	QiDongGuanQingDouJia	24.40
CDS1	IGDB-TZX-8421	SRF300	15.90
CDS1	IGDB-TZX-8431	Hei Nong No.41	20.00
CDS1	IGDB-TZX-844	Ji Nong No.23	22.40
CDS1	IGDB-TZX-8511	JinDou16	20.00
CDS1	IGDB-TZX-8601	Xu Dou No.11	22.00
CDS1	IGDB-TZX-868	FenDou79	17.90
CDS1	IGDB-TZX-8711	JinDa78	21.00
CDS1	IGDB-TZX-878	WanDou24	24.00
CDS1	IGDB-TZX-8901	Liao Dou No.3	18.90
CDS1	IGDB-TZX-9021	Shang Hai Chi Sha Huang Dou	16.60
CDS1	IGDB-TZX-9241	Hei Nong No.69	20.00
CDS1	IGDB-TZX-926	Zhong Huang No.31	20.50
CDS1	IGDB-TZX-956	ZhongHuang67	18.30
CDS1	IGDB-TZX-9611	Zhang Dou No.003	18.00
CDS1	IGDB-TZX-969	LinXianCaoHuangDou	9.00

CDS type	Sample ID	Accessions	HSW (g)
CDS1	IGDB-TZX-9701	Xiong Yue Xiao Li Huang -1	16.20
CDS1	IGDB-TZX-9731	Ken Feng No.15	18.00
CDS1	IGDB-TZX-976	ShuXian205	25.00
CDS2	IGDB-TZX-0111	Si Jiao Qi	12.70
CDS2	IGDB-TZX-0221	Xiao Hei Dou -1	6.00
CDS2	IGDB-TZX-029	Ning Xian Yi Wo Feng	15.90
CDS2	IGDB-TZX-0511	Lei Lei Dou	16.70
CDS2	IGDB-TZX-064	Liao Dou No.15 -1	27.00
CDS2	IGDB-TZX-067	DongShanBaiMaDou	12.00
CDS2	IGDB-TZX-077	Sha Xin Dou	12.30
CDS2	IGDB-TZX-109	FangShanHuangDou	12.50
CDS2	IGDB-TZX-149	XiaoJinYuan	16.30
CDS2	IGDB-TZX-1541	Ji Nf No.58	14.50
CDS2	IGDB-TZX-156	Zhong Huang No.58	20.50
CDS2	IGDB-TZX-241	PI 407801	6.30
CDS2	IGDB-TZX-277	XiangDou4Hao	13.00
CDS2	IGDB-TZX-401	Sable	7.50
CDS2	IGDB-TZX-437	MiYangXiaoZiHuang	10.30
CDS2	IGDB-TZX-439	QianAnYiLiChuan	19.60
CDS2	IGDB-TZX-4611	Jin Yi No.31	18.30
CDS2	IGDB-TZX-4821	Zhong Huang No.39	21.00
CDS2	IGDB-TZX-498	Mi Feng Qiu	12.70
CDS2	IGDB-TZX-5011	LiuShiRiJinHuangDaDou	15.90
CDS2	IGDB-TZX-571	Gnome	16.10
CDS2	IGDB-TZX-588	Da Hong Dou	19.50
CDS2	IGDB-TZX-6121	ShiMianDaDouZi	17.90
CDS2	IGDB-TZX-619	BaiZhenZhu	14.80
CDS2	IGDB-TZX-750	Dairyu Tsurunoko	15.50
CDS2	IGDB-TZX-755	Liao Dou No.31	21.40
CDS2	IGDB-TZX-760	NC 9173	9.50
CDS2	IGDB-TZX-767	HengFengWuDou	13.20
CDS2	IGDB-TZX-7821	ZhongHuang44	23.10
CDS2	IGDB-TZX-8021	Song Jiang Deng Xi Feng Jia	13.60
CDS2	IGDB-TZX-819	HuaTingYangYanQingDou	36.60
CDS2	IGDB-TZX-841	Xiao ma yi dan	4.00
CDS2	IGDB-TZX-8821	ZhongHuang48	22.30
CDS2	IGDB-TZX-891	Kershaw	10.40
CDS2	IGDB-TZX-907	LiuShiTianHuanCang	14.10
CDS2	IGDB-TZX-912	Ogden	11.40
CDS2	IGDB-TZX-967	YanTianQingPiDou	13.50
CDS2	IGDB-TZX-9801	Hong Da Dou	16.50
CDS2	IGDB-TZX-9821	ZhongHuang49	20.20
CDS2	IGDB-TZX-990	Shakujo	12.00
CDS2	IGDB-TZX-9901	Tie Feng No.22	16.00

HSW, 100-seed weight. The sequence variation in the CDS region of *Glyma.19G143300* and the phenotypic data of 100-seed weight was download from the database (http://www.mbkbase.org/soybean).

References

Li N, Xu R, Li YH (2019) Molecular networks of seed size control in plants. Annu Rev Plant Biol 70:435-463
